# Butler Matrix Based on Substrate Integrated Waveguide Without Crossover and Phase Shifter for Millimeter Waves Applications

**DOI:** 10.12688/f1000research.168318.1

**Published:** 2025-09-23

**Authors:** Nawres Nayyef, Kim Geok Tan, Hussam Keriee, Zahriladha Zakaria, Chia Pao Liew, Chockalingam Aravind Vaithilingam

**Affiliations:** 1 Multimedia University Faculty of Engineering and Technology, Ayer Keroh, Malacca, Malaysia; 2 Centre for Advanced Analytics, CoE for Artificial intelligence, Multimedia University, Ayer Keroh, Malaysia; 3 Ministry of Higher Education and Scientific Research, Baghdad, Iraq; 4 Faculty of Electronics and Computer Engineering, Universiti Teknikal Malaysia Melaka, Durian Tunggal, Malacca, Malaysia; 5 School of Energy and Chemical Engineering, Xiamen University Malaysia, Sepang, Selangor, Malaysia; 6 Taylor's University School of Engineering, Subang Jaya, Selangor, Malaysia

**Keywords:** Butler matrix, Hybrid coupler, Vias, Substrate integrated waveguide, 5G

## Abstract

**Background:**

A novel low-loss 4 × 4 Butler Matrix structure for 26 GHz operation is designed in this study. The proposed 4 × 4 antenna Butler matrix comprises an inscribed structure, which makes it fundamentally different from traditional beam-forming networks in 5G 26-GHz applications.

**Method:**

This novel Butler matrix comprises four hybrid couplers without crossovers or phase shifters. To reduce the size, magnitude, and phase difference, and to increase the bandwidth, interdigital and non-metallic vias are used in the coupler design. The performance of the proposed matrix was simulated using the CST software and implemented on a Rogers 8085 substrate. It was also interfaced with four substrate-integrated waveguide slot antennas to demonstrate its distinct beam scanning capability.

**Results:**

The matrix yielded an error magnitude loss of 1 dB and a phase error of 1.7° with a broad operational bandwidth of 3 GHz. The measured coupling factor of the 4 × 4 Butler matrix at 26 GHz is -6 ±1 dB at the output. The measured results validate that four phase-scanning states at -14.3°, 14.88°, -32.21°, and 31.33° can be realized with return losses of less than -10 dB.

**Conclusions:**

The proposed design achieves a compact, efficient, and low-loss beamforming solution suitable for 5G applications at 26 GHz, demonstrating distinct scanning features without the need for crossovers or phase shifters.

## Introduction

Over the past few years, phased arrays and beamforming have become essential elements in multiple fields, particularly in 5G wireless communication systems. An antenna array is typically controlled by a feeding network that controls the phase shift and amplitude of the respective antenna elements. ^1^ The antenna array is most easily articulated in these embodiments because the radiation beam is steered by the antenna array towards the selected position. This feature gives rise to three different types of feed networks for the control of antenna arrays: parallel-feed networks, series-feed networks, and matrix-based networks. ^2^ Wide-tuning phase shifters are required for both parallel and series feed networks, and they restrict the capability of the antenna array to simultaneously form more than one beam at the same time. However, this was not the case for matrix feed networks, which had hybrid couplers, crossover, and a phase shifter network. ^3^ In addition, matrix networks have the special property that multiple beams can be produced simultaneously. Different matrix feeding networks have been designed, such as the widely used Butler, Blass, and Nolen matrices. ^4– 6^ The Butler matrix is well known for its symmetric design with an equal number of inputs and outputs. Equal-magnitude signals with progressive phase differences are emitted from the output ports ^7^ of each input port, and the simultaneous radiation of the two beams can be accomplished. The Butler matrix has been widely investigated for its low loss, ^8^ multiband, ^9^ wideband, ^10^ small size, ^11^ and millimeter-wave operating ranges. ^12^ Furthermore, studies on symmetric structures at two scales, including the 4× 4 ^13^ and 8× 8 networks, have also been performed. ^14^ Later studies employed the Butler matrix for non-symmetric structures, such as the 2 × 4 network, ^15^ 3 × 4 network, ^16^ and 4 × 8 network. ^17^ The Blass matrix is a cross-over free full-rank matrix with half as many input and output ports as the Butler matrix, but load terminations in the Blass matrix substitute for the crossovers of the Butler matrix. There are structural limitations of the Blass matrix that could lead to partial signal flow towards the termination loads, which causes the efficiency to be less than that of the Butler matrix. ^5^ To overcome this drawback, improvements in the Butler matrix have been suggested, focusing on a reduction in the number of components and element footprints. However, for lower 5G bands such as 26 GHz, beamforming components (e.g., couplers, phase shifters, and antenna elements) have limited flexibility and high signal loss. ^18^ An early beamforming network was introduced in Ref. 19 where it incorporated a Butler matrix algorithm in combination with a 90° coupler. Another development in beamforming networks was considered in Ref. 20, which reports on a series-fed matrix design that suppresses sidelobes at 28 GHz. In Ref. 21, a microstrip beamforming system was presented for multiband operations at 26, 28, and 30 GHz. The coupler used in this configuration was H-type with a short slot. One recent example is in Ref. 22, where the work is at 28 GHz with a 4 × 4 Butler matrix structure for a 2-D beamforming network that uses three couplers and three phase shifters. Another example of a compact form factor is the 4 × 4 beamforming network at 28 GHz, which was designed in Ref. 23 using lumped element couplers to alleviate the matrix size. In addition, a 4 × 4 beamforming network using 90-degree coupler was presented in Refs. 24 and 25 for 28 GHz, with four Substrate Integrated Waveguide (SIW) couplers, one crossover, and two phase shifters. Unfortunately, this structure is large in size with a very high IL and phase difference, which consequently results in size and phase error difficulties in these prior realizations at such increased 5G frequencies. In this paper, a novel 4 × 4 Butler matrix with low loss, compact size, small amplitude, and phase error across the band is presented, based on a normal and unequal separation vector structure along the waveguide walls. The remainder of this paper is organized as follows. First, the topology of the design and equations of the Butler matrix and its variants, as well as those of the design of couplers, are studied. Second, a comprehensive 4 × 4 Butler matrix approach is presented with flexible performance. Subsequently, the measurement results of the proposed 4 × 4 Butler matrix are systematically compared and discussed with respect to simulations and some works presented in the literature. Finally, the major findings are summarized. Taken together, this novel Butler matrix structure is promising for improving beamforming networks of 5G wireless communication systems.

## Method – Design of beamforming

**Figure 1.**
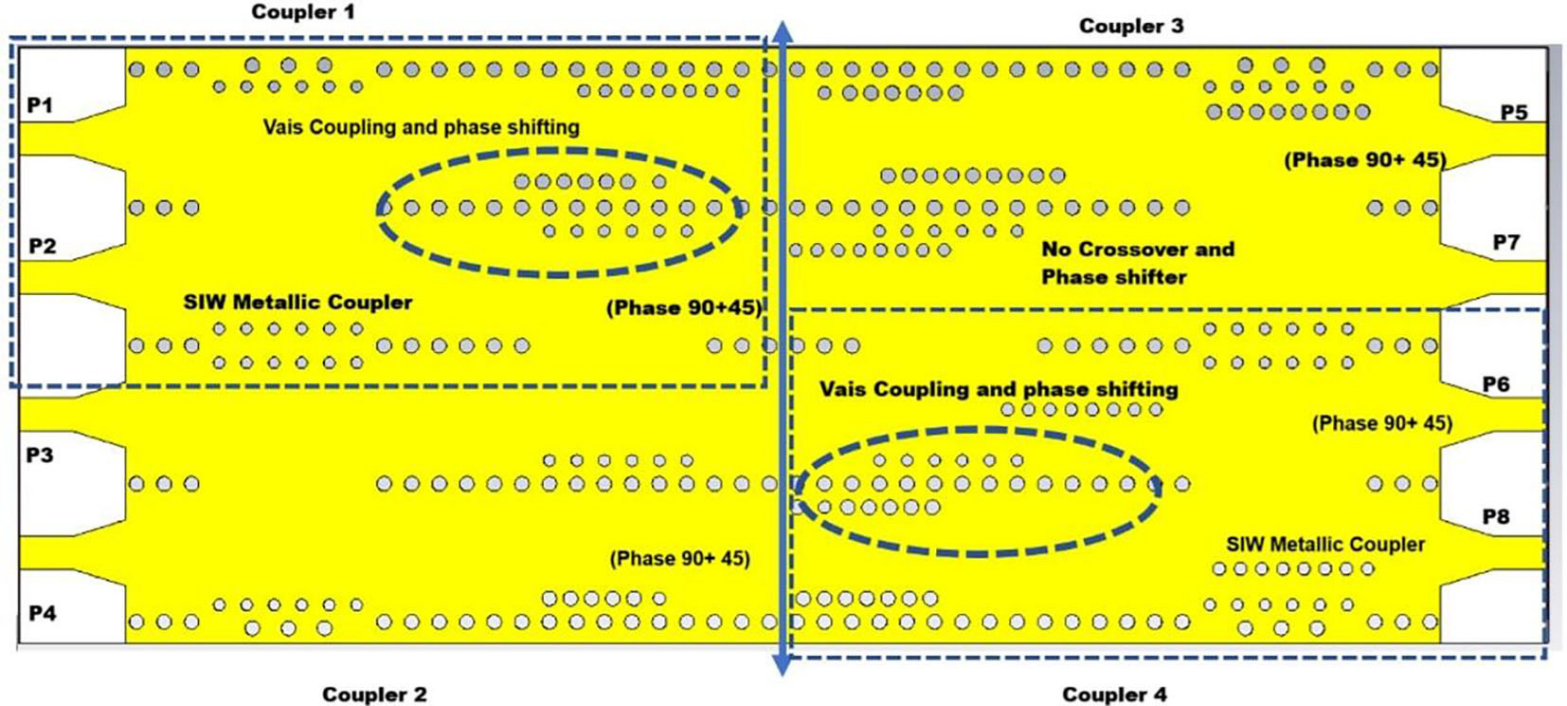
The structure of 4 × 4 SIW Butler Matrix.

Figure 1 presents the structure of the 4 × 4 Butler matrix, where the four input ports are defined as (P1, P2, P3, and P4) and the four output ports are represented as (P5, P6, P7, and P8). The Butler matrix presented in Figure 1 contains only four hybrid couplers. All couplers have a single coupling constant that is distributed along the matrix equally between the lower and upper parts. To maintain edge-to-edge uniformity of signal levels (for the output of Butler matrix) and signal integrity of lines, the coupling ratios of (C1) and (C2) were selected. Hence, the coupling ratio of the couplers at the Butler matrix outputs can be expressed as Ref. 22.

**Figure 2.**
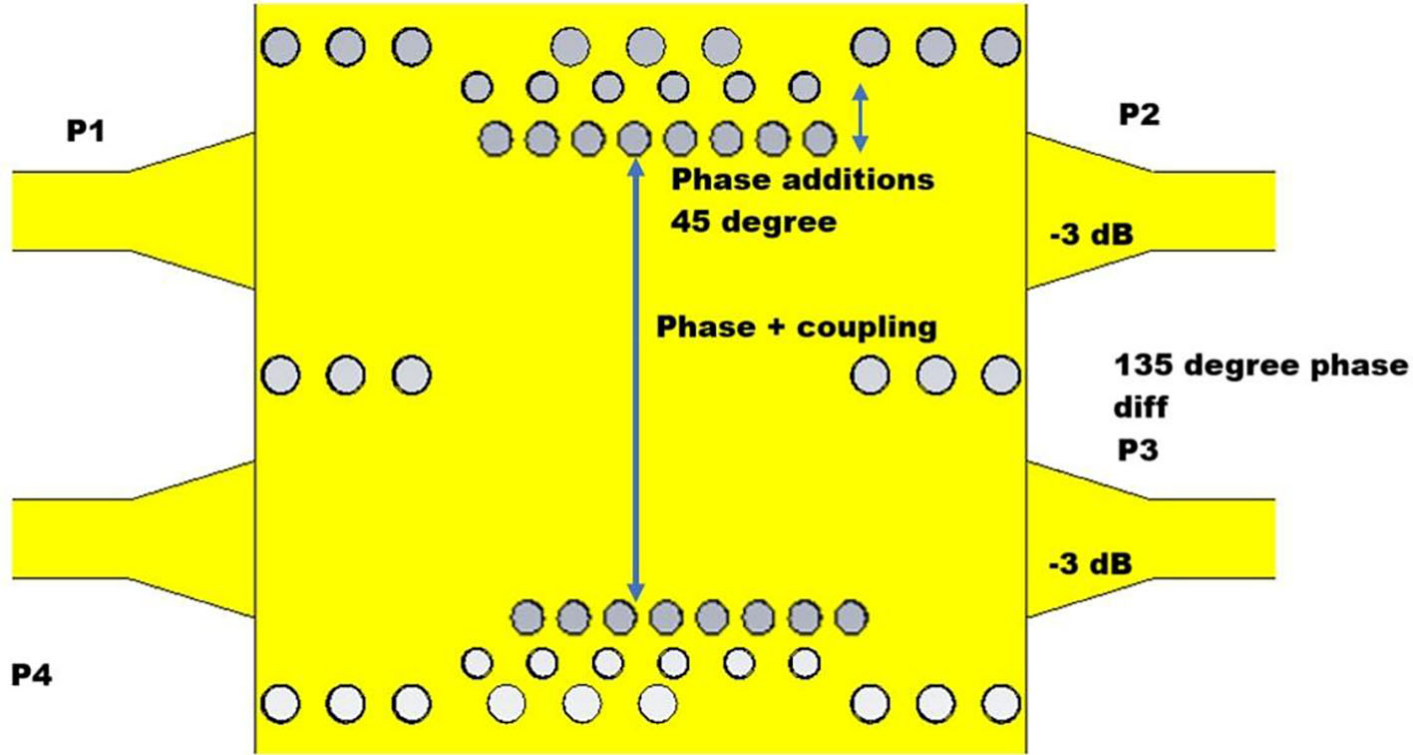
The structure of SIW Hybrid coupler with phase difference of 135 degree.

The schematic structure of the two-section VAI hybrid coupler used for the speed demonstration with a center frequency of 26 GHz is illustrated in Figure 2. The coupler is designed to have a symmetric four-port structure (P1–P4) and is based on a via-based internal coupler structure for coupling and phase modulation. The proposed power splitter is constructed using substrate-integrated waveguide (SIW) technology, the two power-dividing arms of which are synthesized by electromagnetic coupling and phase manipulations to achieve the desired characteristics. The design embodies 50 Ω quarter-wavelength transmission lines as the basic structure of the coupler arms. The arms are realized by a set of carefully optimized strip-line widths based on the microwave design equations found in Refs. 24 and 25. Rows, which act as artificial magnetic conductors, are mainly utilized to confine electromagnetic fields but also affect impedance matching and phase response. This is evident in Figure 2, which also shows the signal flow from port to port. The input power at port P1 is equally divided into ports P2 and P3, with-3 dB at each port. In addition, the coupler introduces a well-defined phase relationship, which provides 45° and constructive phase coupling at the central transmission axis, whereas the phase shift difference between the output ports is 135°. This phase shift is essential for many beamforming systems, such as the Butler matrix in 5G phased arrays. To widen the operating band, an arm-shaped structure was used for each coupling section. The modified arms act as impedance transformers to make the required impedance transitions (Z _1_, Z _2_, Z _3_) over the band. The design equations are formulated in closed form as related to even- and odd-mode analyses to obtain low reflection and high isolation between ports. In general, the design exhibits good engineering accuracy, particularly with respect to impedance tuning, phase control, and coupling balance its due regard. These hybrid couplers are important in millimeter-wave transceivers, where the architecture, efficiency, and bandwidth require compactness, and wideband performance is achieved by providing degrees of freedom for phase control and power division in the physical layout. When a single is applied to port P1, the power is divided equally between ports P2 and P3, with a loss of approximately 3 dB. The signal path includes phase-adding means: in the vertical direction, it is a 45° phase adder, while the output ports P2 and P3 retain their 135° phase difference, which is essential for modern millimeter-wave beamforming systems where, for example, the Butler matrix is implemented.

**Figure 3.**
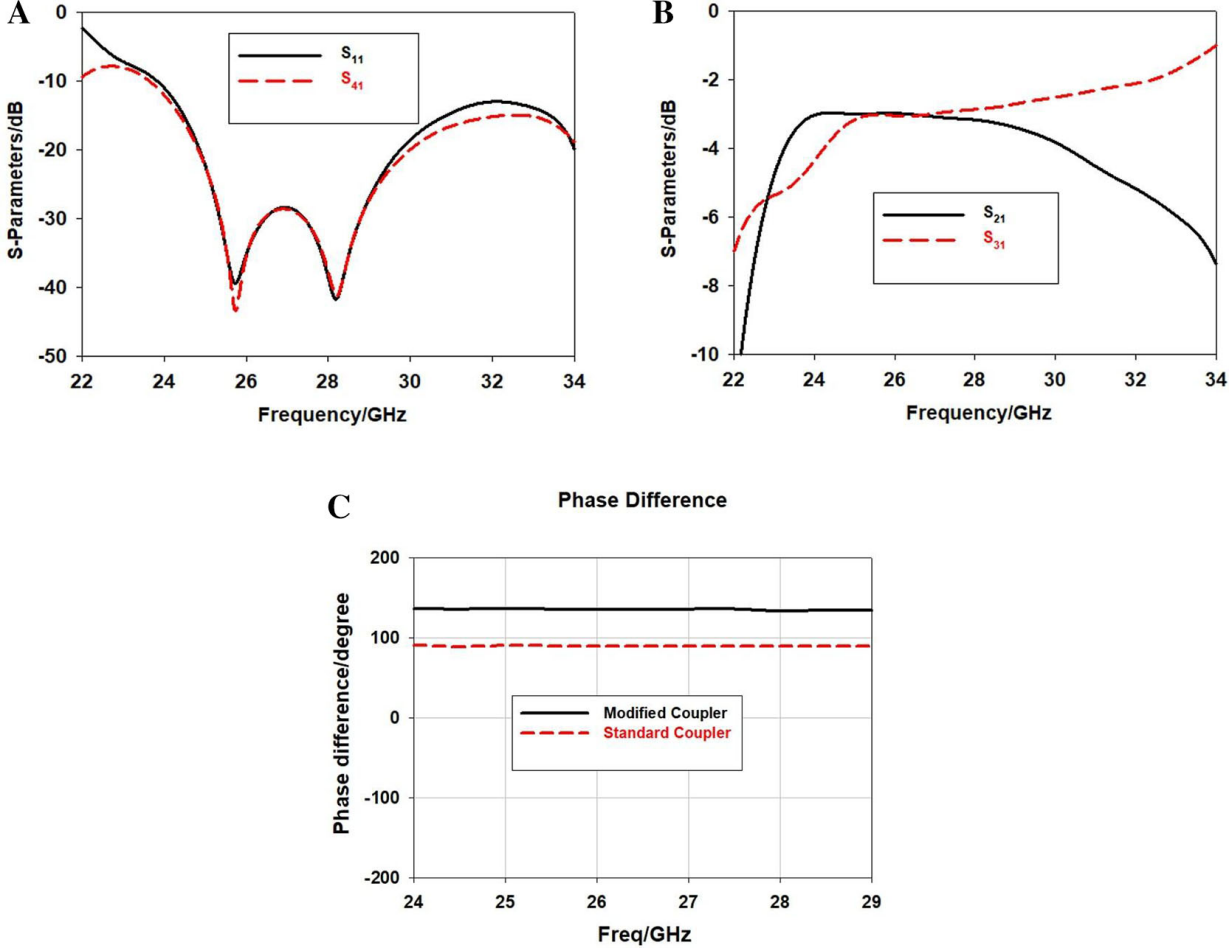
Simulated response for designed hybrid coupler. (A) Return loss and isolation, (B) Output power, (C) Phase difference vs standard coupler.

The response of the coupler was characterized using numerical simulations based on full-wave electromagnetic equations. The obtained S-parameters indicate the operational bandwidth, matching properties, and isolation performance of the coupler and are depicted in Figure 3. As shown in Figure 3(A), a sharp dip below -30 dB in | *S*_11_| was observed at a center frequency of 26 GHz, indicating a good impedance-matching Doppler radar. The isolation from port P1 to isolated port P4 ( $S_{41}$ ) follows a similar behavior, achieving values less than -30 dB at resonance and a minimum close to -40 dB. These findings verify the effective suppression of unwanted reflections and high isolation between noncoupled ports, which is an essential figure of merit for hybrid couplers. The coupling levels from input P1 to output ports P2 ( $S_{21}$ ) and P3 ( $S_{31}$ ) are well suppressed, as shown in Figure 3(B). At 26 GHz, both $S_{21}$ and $S_{31}$ meet at about -3.1 dB, confirming the desired equal power splitting. Furthermore, making a power splitter at a power splitting range of -3 dB to -4.5 dB over a 5-GHz frequency range (from 24.5 to 29.5 GHz), that is, operation bandwidth is also realized. As shown in Figure 3(C), the phase difference between ports P2 and P3 was 135°, not the standard 90°, which reflects a deliberate design choice. This enhancement of the phase difference enables the coupler to integrate both the coupling and phase-shifting functions into a single device, thereby combining the roles of a hybrid coupler and phase shifter.

**Figure 4.**
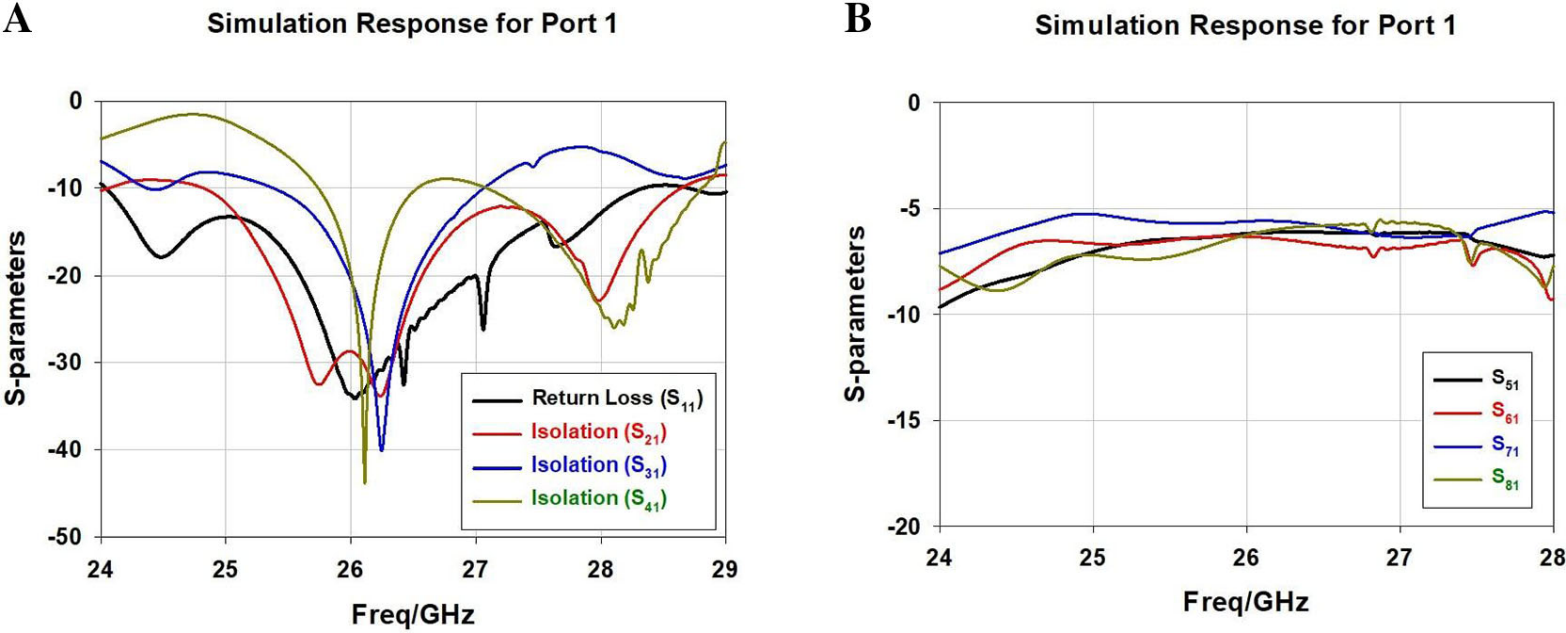
Simulated Port 1 of BM. (A) Return loss and isolated ports, (B) Output ports.

**Figure 5.**
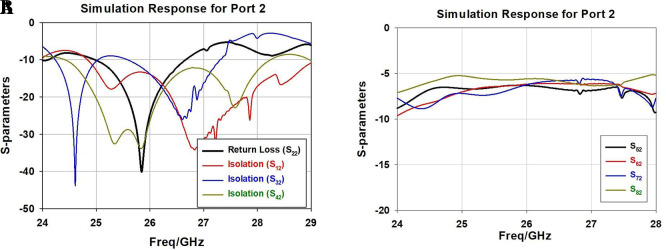
Simulated Port 2 of BM. (A) Return loss and isolated ports, (B) Output ports.

**Figure 6.**
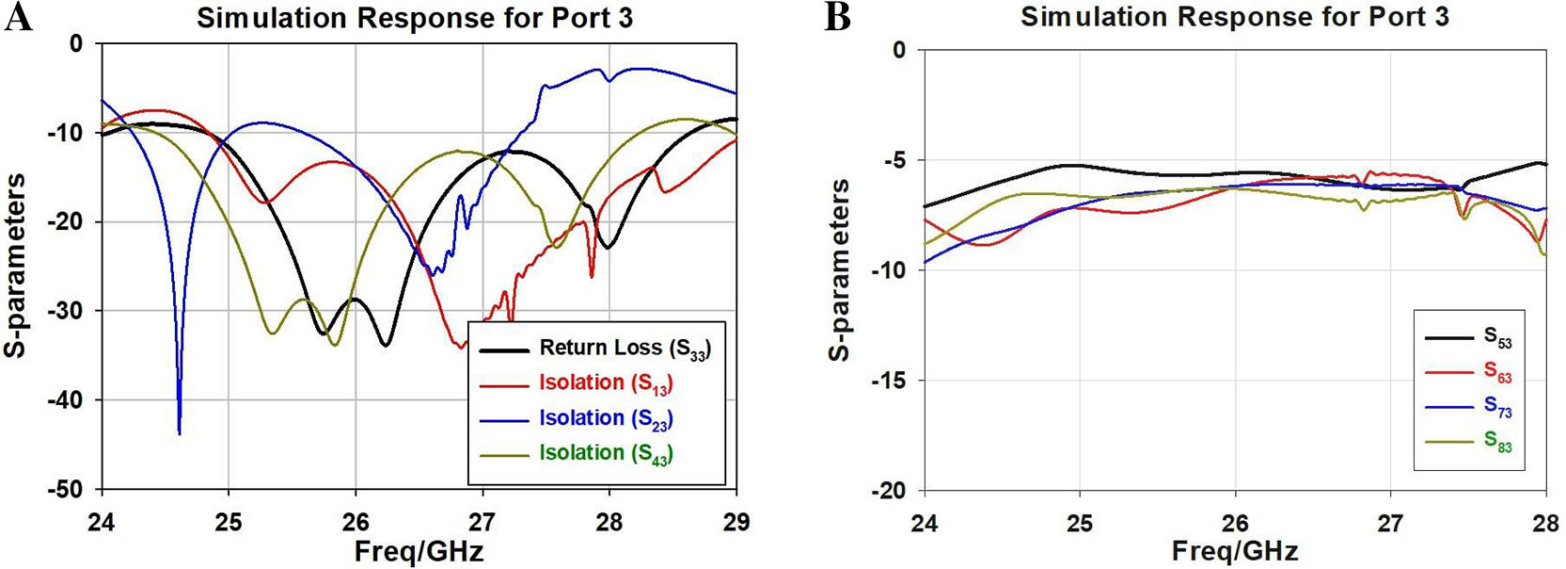
Simulated Port 3 of BM. (A) Return loss and isolated ports, (B) Output ports.

**Figure 7.**
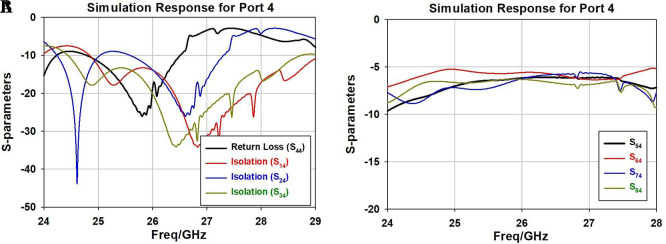
Simulated Port 4 of BM. (A) Return loss and isolated ports, (B) Output ports.

As mentioned earlier, Figure 1 shows the implementation of a 4 × 4 Butler matrix design and the assembly of four couplers. A simulation using Computer Simulation Technology (CST) was carried out to prove the feasibility of the 4 × 4 Butler matrix. The S-parameters are linked to the return loss, isolation, and transmission coefficients, as shown in Figure 4(A), 5(A), 6(A), and 7(A), respectively. As shown in Figure 4, the return loss of input port 1 shows acceptable results in all bands and is below -10 Db. More precisely, at 26 GHz, the measured return loss values were *S*_11_ = −20:4 dB, *S*_21_ = −18:7 dB, and *S*_31_ = −22:9 dB, yielding a fractional bandwidth of 60% over all input ports. This demonstrates that good isolation was achieved for all the ports. Figure 4(B) also shows the transmission coefficients when port 1 is powered on, it produces S _51_ = -5.8 dB, S _61_ = -6.12 dB, S _71_ = -6.12 dB > S _61_, and S _81_ = -6.5 dB. These results indicate that the power is distributed equally in the outputs if Port 1 is excited. Thus, the calculated S-parameter results corresponded to the design specifications of the 4 × 4 Butler matrix. The input return loss at input port 2 is superior and less than -10 dB. At 26 GHz, for example, the return loss measurements read *S*_22_ = -20.4 dB, *S*_12_ = -18.7 dB and *S*_13_ = -22.9 dB, which encompasses a fractional bandwidth of 60% for all of the input ports. This shows that the isolation between the ports is good. Figure also shows the behaviour of the transmission coefficients when Port 2 is on. As shown in Figure 5(B) The measured values are *S*_52_ =-6.8 dB, *S*_62_ = -6.35 dB, *S*_72_= -6.54 dB, and *S*_82_ = -6.55 dB. This implies that equal power is distributed to the outputs with excitation at port 2. The return loss of input port 3 is shown in Figure 6(A), with good performance below -10 dB. Return losses values of *S*_33_ = -25.5 dB, *S*_23_ = -20.7 dB, and *S*_43_ = -24.7 dB at 26 GHz and a fractional bandwidth of 60% are obtained over all input ports This indicates that proper isolation was achieved at each port. The transmission coefficients upon the activation of port 3 are shown in Figure 6(B). The measured values are *S*_53_ = 6.2 dB, *S*_63_ = 5.85 dB, *S*_73_ = 6.22 dB and *S*_83_ = 6.75 dB which implies that power equally distributes among the ports when port 3 is excited. Figure 7 shows the return loss behaviour at input port 4, where excellent performance is observed with a value of less than -10 dB. Remarkably, at 26 GHz, the return loss measurements are *S*_44_ = -24.7 dB, *S*_43_ = -22.8 dB, and *S*_42_ =- 22.6 dB, respectively, which again constitute a 60% fractional bandwidth for all input ports. This means that strong isolation is reached at each port. The transmission coefficient responses upon the activation of port 4 are shown in Figure 7(B). The measured *S*_53_ = -6.8 dB, *S*_63_= -5.47 dB, *S*_73_ = -6.65 dB, and *S*_83_ = -6.52 dB, suggest that when port 4 is involved, the output power is evenly divided.

**Figure 8.**
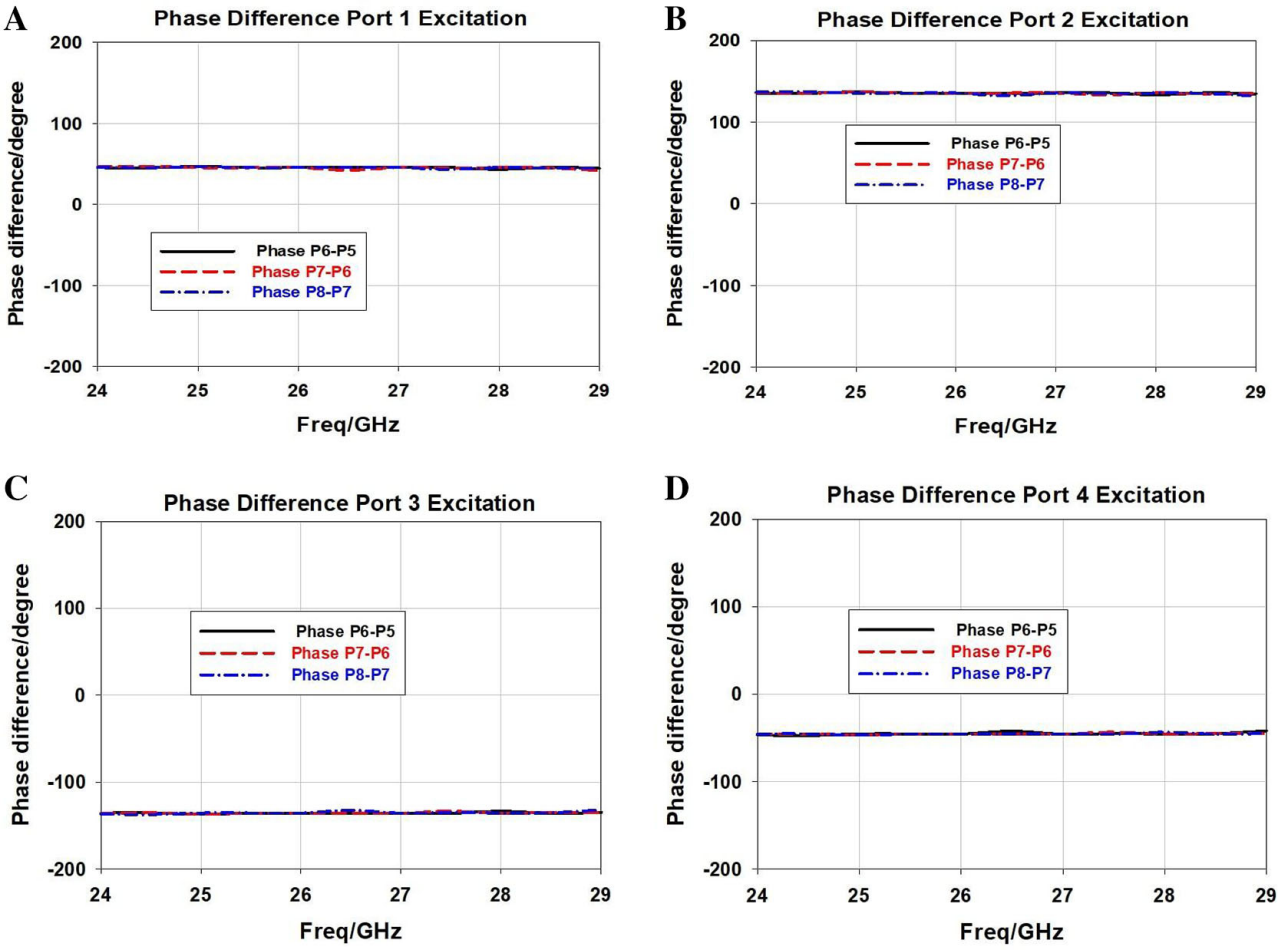
Simulated phase differences of proposed BM. (A) Port 1, (B) Port 2, (C) Port 3, (D) Port 4.

The simulated phase shifts of the output ports with excitation from Port 1 are shown in Figure 8(A). In particular, when port 1 is actuated, the phase dispersion of the outputs at ports 5, 6, 7, and 8 is 45.6°. By turning on port 2, a phase difference of 133.6° can be observed, as shown in Figure 8(B). Note from the accompanying figure that all outputs (1, 2, and 3) have the correct phase of excitation when the signal from port 2 is set. The simulated phase shifts for the output ports under excitation at port 3 are shown in Figure 8(C) together with the phase differences for the four output ports of the 4 × 4 Butler matrix. It can also be found that when port 3 is excited, the simulated phase difference between the outputs (i.e., ports 5, 6, 7, and 8) is -133.23°, as shown in Figure 8(C). -45.6° is the phase difference when port 4 is excited, as shown in Figure 8(D).

## Results and Discussion

**Figure 9.**
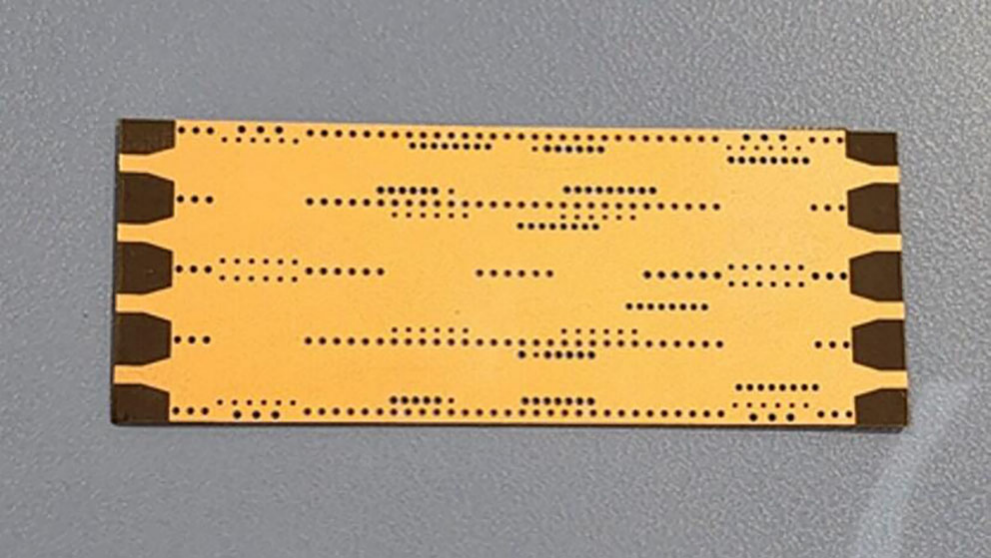
Fabricated 4 × 4 SIW BM.

**Figure 10.**
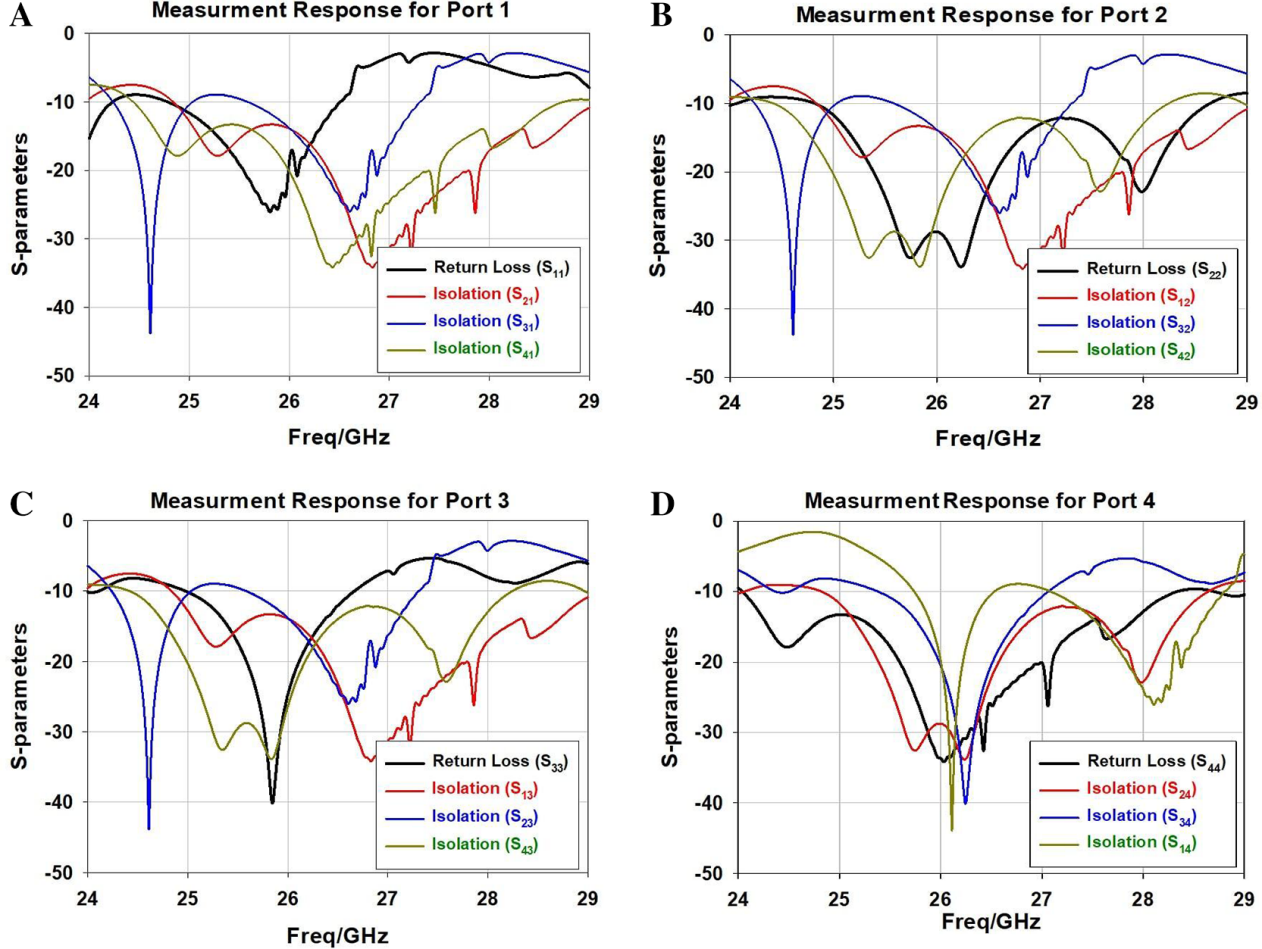
Measured results of SIW BM. (A) Port 1, (B) Port 2, (C) Port 3 and (D) Port 4.

The suggested 4 × 4 Butler matrix design was realized on a Roger substrate with a thickness of *h* = 0.508 mm and relative permittivity of *ε*_r_ = 2.2. Figure 9 shows the manufactured 4 × 4 Butler matrix. The S-parameter tests were performed using a Keysight (Agilent Technologies) field forecast vector network analyzer (VNA) (N9925A), two cables, and six referent dummy loads. The efficiency of the 4 × 4 Butler matrix hardware is demonstrated by its *S*-parameters, as seen in the simulation results shown in Figure 10.

The measured return loss and isolation from the port 1 excitation are plotted in Figure 10(A). The measured *S*_11_ is −20 dB at 26 GHz, and the wideband response has a 2 GHz percentage of 60 FBW of operation. The results of these simulations were compared with the measurements. Furthermore, the isolation of ports 2, 3, and 4 at all reconfigurable states is satisfactory and remains less than -10 dB over the desired band, as illustrated in Figure 10(A). The measured transmission coefficients in Figure 10(A) are *S*_51_ = −6.5 dB, *S*_61_ = −6.69 dB, *S*_71_ = −6.69 dB, *S*_81_ = −7.2 dB, which shows an amplitude loss of −2.2 dB. Therefore, a low insertion loss was obtained for the port 1 excitation. The return loss and isolation of the port 2 excitation are shown in Figure 10 (B). In particular, *S*_22_ is measured at -15 dB and 26 GHz with a wideband of 2.4 GHz to maintain a 60% FBW, and is set for comparison with the simulated results. On the other hand, the remaining isolations (ports 1, 3, and 4) also perform well, with isolations in the range of −10 dB over the desired frequency band. The transmission coefficients of Figure 10(B) are given as *S*_52_ = -7.5 dB, *S*_62_ = -7.69 dB, *S*_72_ = -6.49 dB, and *S*_82_ = -6.2 dB, indicating amplitude attenuation of -1.5 db. Hence, for the port 2 drive, a low insertion loss is found. The simulated return loss and isolation at the port 3 excitation are shown in Figure 10(C). The measured −18.6 dB *S*_33_ at 26 GHz, together with the wideband characteristic (26 GHz, 55% FBW), is compared with the simulated results. The measured isolations from ports 1, 2, and 4 were well maintained at less than even -10 dB over the designed bandwidth, as depicted in Figure 10(C). The measured transmission coefficients in Figure 10(C) are *S*_53_= -6.58 dB, *S*_63_ = -6.49 dB, *S*_73_ = -7.49 dB and *S*_83_ = -7.3 dB with magnitude loss of –1.6 dB. Accordingly, in the port 3 excitation, a low insertion loss was attained. The measured return loss and isolation for the port 4 excitation are shown in Figure 10(D). This measured *S*_44_= -19.7 dB at 26 GHz over a wideband characteristic of 4 GHz (60% FBW) is then compared to simulated results. In contrast, the measured isolation from ports 1, 2, and 3 exhibits a good performance of lower than -10 dB over the desired bandwidth, as illustrated in Figure 10(D). From the Figure 10(D), the measured transmission coefficients are -7.58 dB ( *S*_54_), -6.87 dB ( *S*_64_), -6.77 dB ( *S*_74_) and –7.58 dB ( *S*_84_), with a magnitude loss of -1.85 dB. Thus, the insertion loss at the port 4 excitation was also low.

**Figure 11.**
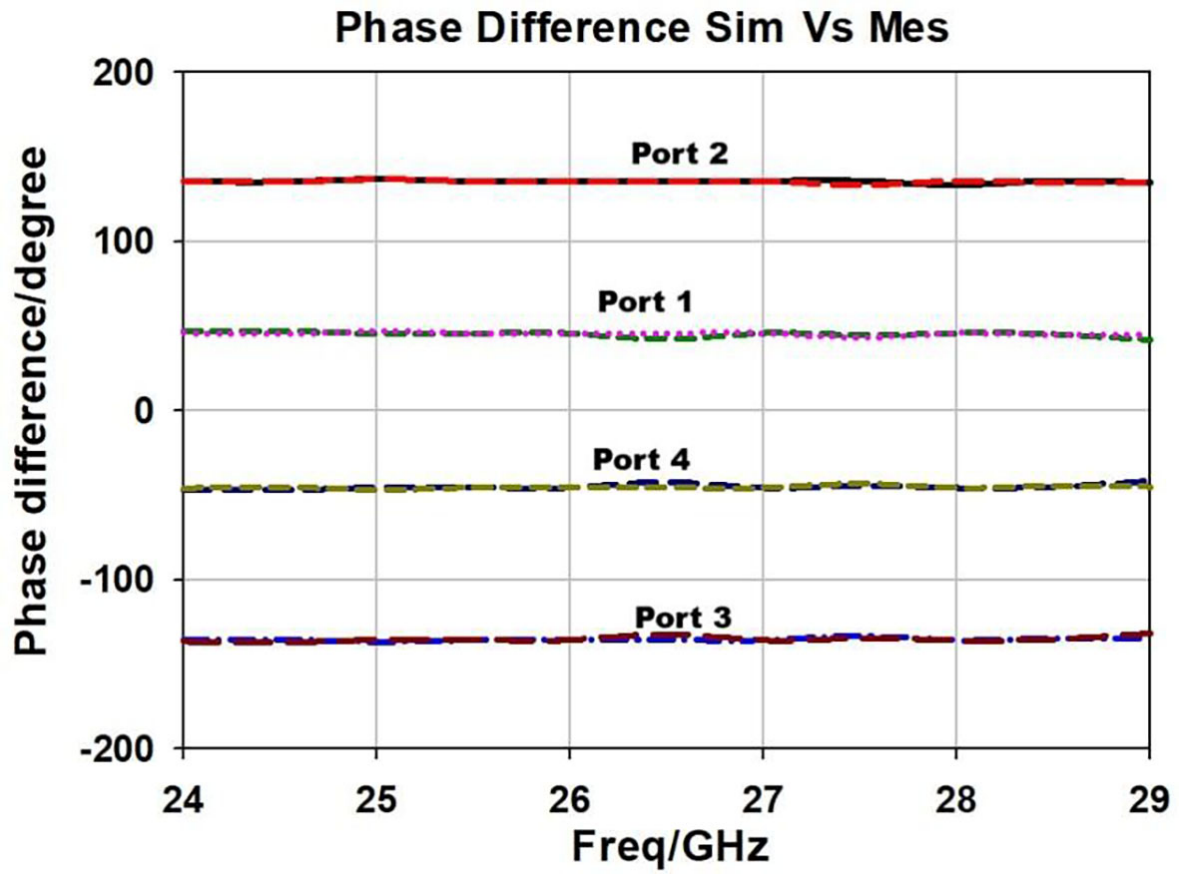
Measured vs simulated phase difference at 26 GHz.

The measured phase difference of the outputs when all the input ports are active is shown in Figure 11. When compared with the simulated phase differences, it was observed that the measured phase difference at the port 1 excitation was 131° with a phase error equal to 1°. When the excitation was performed from ports 2 and 3, the phase differences were 46.05° and -46.04° with phase errors of 1.05° and -1.04° respectively. The measured phase difference at port 4 was − -134° with a phase error of 2°. Therefore, the proposed Butler matrix has a tunable phase difference at the output ports, with an average phase error of 2° at the low-frequency stage.

**Figure 12.**
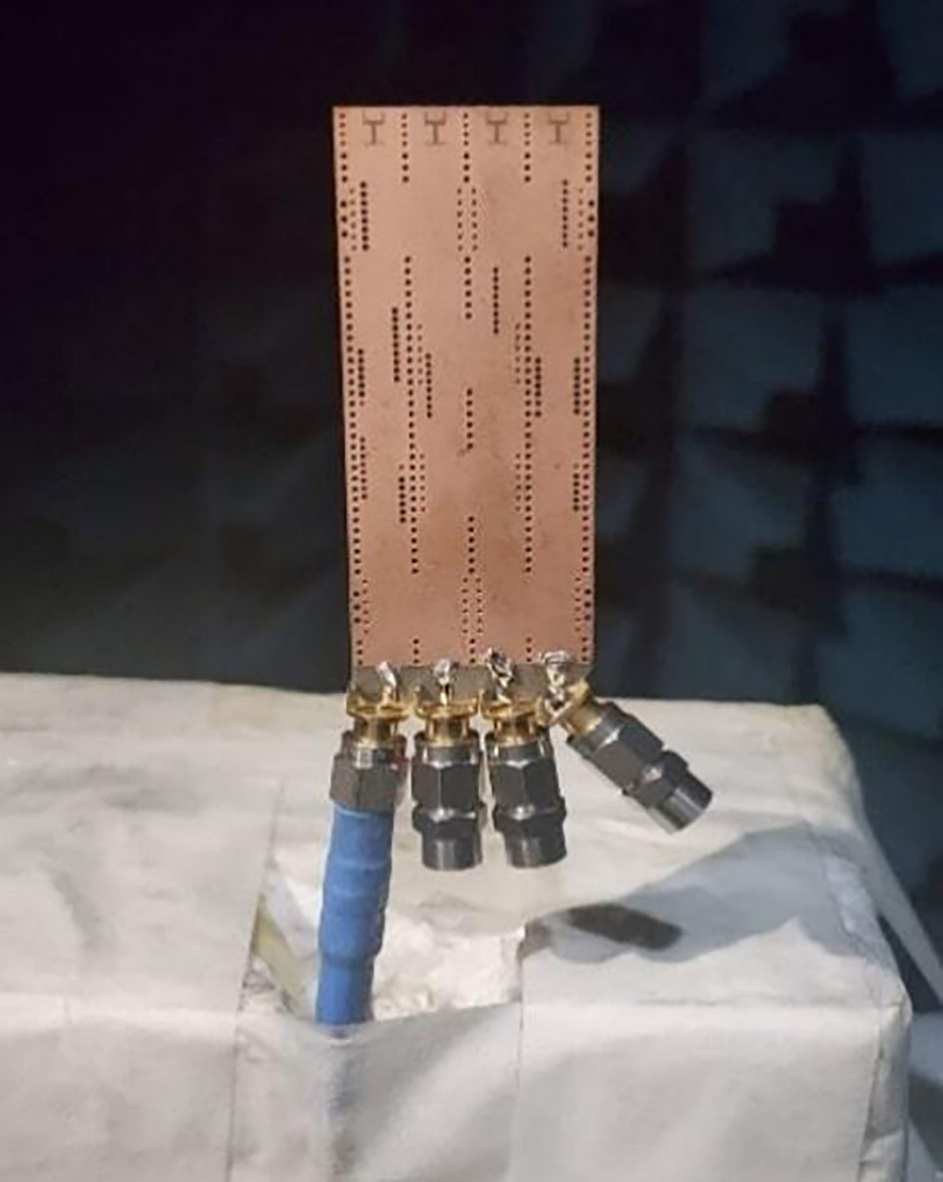
The fabricated beamforming network.

**Figure 13.**
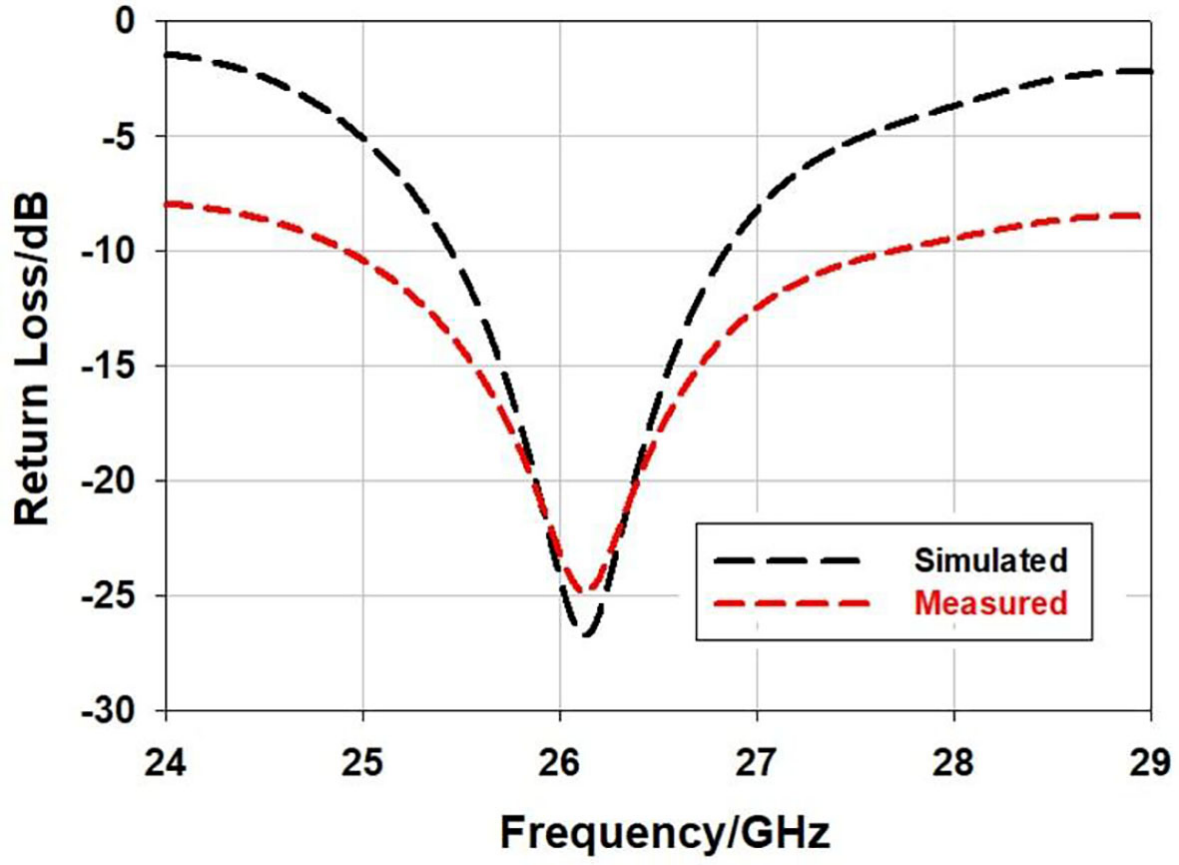
The measured vs simulated return loss of beamforming.

**Figure 14.**
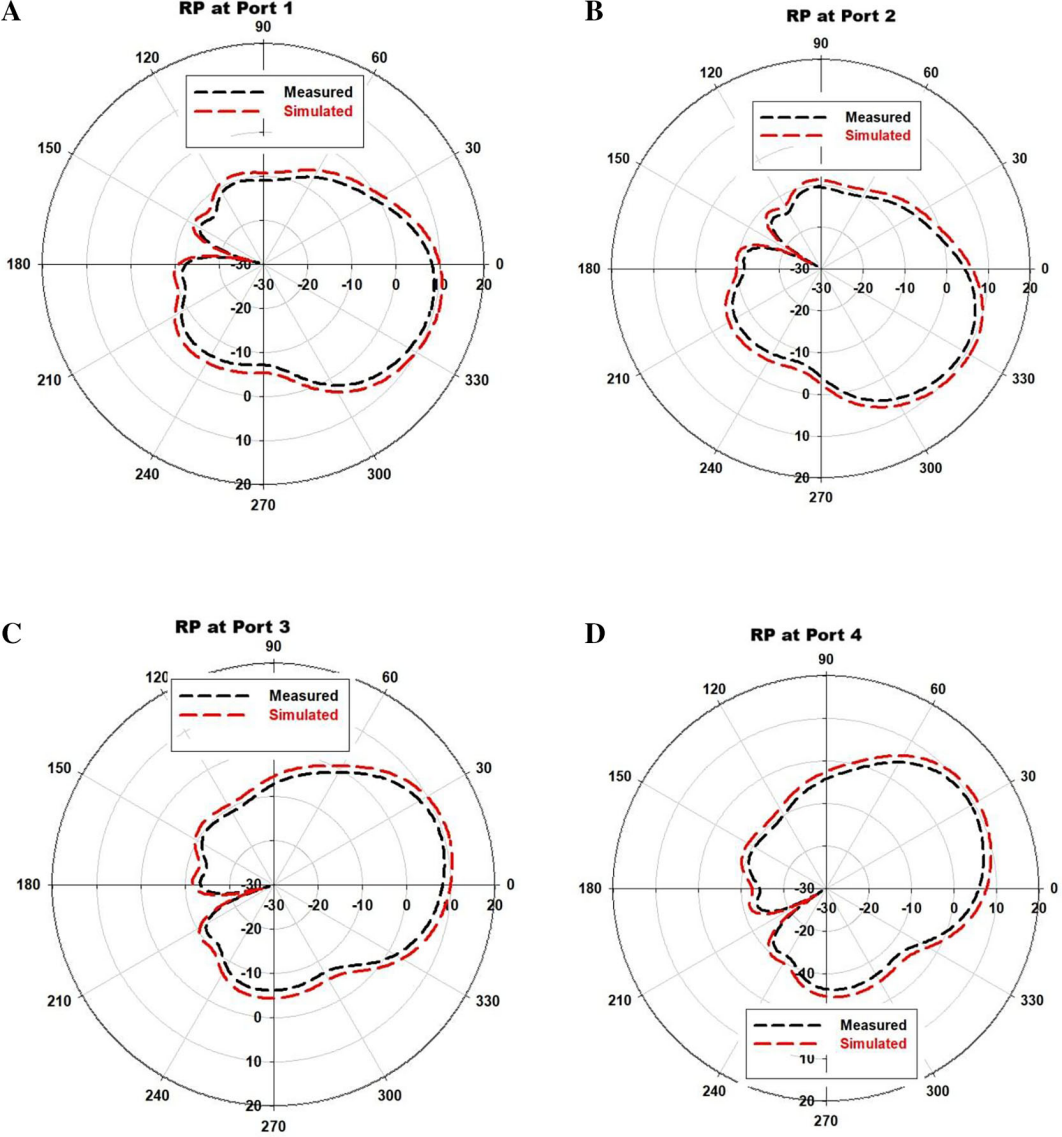
The measured vs simulated RP at 26 GHz. (A) Port 1, (B) Port 2, (C) Port 3, (D) Port 4.

The prototype of the proposed antenna beamforming network with a Butler matrix network is shown in Figure 12. The simulated return loss of the proposed beamforming network is illustrated in Figure 13. At 26 GHz, the return loss for all excited ports is < −10 dB. This suggests that the beamforming feeding network performed well at the desired bandwidth. Nonetheless, at ports 2 and 3, a decay in the signal was detected because of the mismatch between the connectors and channels, as well as the losses of the cable. The bandwidth was approximately 2.8 GHz, which suggests that the performance of the beamformer is highly resonant at various frequencies. The measured and simulated radiation patterns for the designed BM in the SIW are shown in Figures 14(A), 14(B), 14(C) and 14(D). Owing to ±31.23° for all ports, 9 dB beams were obtained at ±14.43° and ±11 dB at all ports. In summary, a low gain loss (within 3 dB) with a low phase error of 1.8° at port 2 was obtained. A comparison with other similar-frequency designs is presented in Table 1. Therefore, the proposed beamforming network met the design specifications well and had a better performance than state-of-the-art devices with low loss and low profile and phase error at 26 GHz.

**Table 1.** Comparison of this work with other related research work.

Ref	Technology	Freq.	Configuration	Losses	Phase error	Size (mm ^2^) (L × W)
^22^/2021	Waveguide	28 GHz	4 × 4 BM Standard	3.4 dB	4°	130 × 66 mm ^2^
^23^/2022	Microstrip	28-32 GHz	6 × 6 BM Standard	8.5 dB	10°	130 × 38 mm ^2^
^21^/2023	SIW	26 GHz	4 × 4 BM Standard	5.3 dB	6.7°	95 × 30 mm ^2^
^24^ /2024	SIW	28 GHz	4 × 4 BM Standard	6 dB	5°	93 × 42 mm ^2^
^25^/2024	SIW	26 GHz	4 × 4 BM Standard	4.8 dB	7°	100 × 35 mm ^2^
This work/2025	SIW	26 GHz	4 × 4 BM couplers	1.4 dB	1.8°	74 × 28 mm ^2^

## Conclusion

A 4 × 4 Butler beamforming matrix with a low-loss wideband at 26 GHz for 5G mobile proposed to produce one type of 4 × 4 application is shown to produce a distinct sequential phase difference available in the output ports. The fabricated 4 × 4 Butler matrix was designed with four 3-dB couplers to achieve substantial size reduction. There is a good agreement between the measurement and simulation results to verify the final performance of the design. A high performance 1.4 dB low loss magnitude error, 1.8° phase error and a 2.8 GHz 1 dB bandwidth are achieved. The suggested 4 × 4 antenna Butler matrix has a novel method for BFN designs for 26 GHz 5G applications along with the presented properties.

## Data Availability

Zenodo. Experimental data for “Butler Matrix Based on Substrate Integrated Waveguide Without Crossover and Phase Shifter for Millimeter Waves Applications”. DOI: https://doi.org/10.5281/zenodo.17076167. ^26^ This project contains the following underlying data:
• Experimental Data (Nawras, Coupler and BM).xlsx Data are available under the terms of the Creative Commons Attribution 4.0 International license (CC-BY 4.0).

## References

[1] Ojaroudiparchin N Shen M Zhang S : A Switchable 3-D-Coverage-Phased Array Antenna Package for 5G Mobile Terminals. *IEEE Antennas Wirel. Propag. Lett.* 2016;15(c):1747–1750. 10.1109/LAWP.2016.2532607

[2] Moon SM Yun S Yom IB : Phased Array Shaped-Beam Satellite Antenna with Boosted-Beam Control. *IEEE Trans. Antennas Propag.* 2019;67(12):7633–7636. 10.1109/TAP.2019.2930129

[3] Vallappil AK Rahim MKA Khawaja BA : Butler Matrix Based Beamforming Net-works For Phased Array Antenna Systems: A Comprehensive Review and Future Directions For 5G Applications. *IEEE Access.* 2020;9:3970–3987. 10.1109/ACCESS.2020.3047696

[4] Dyab WM Sakr AA Wu K : Dually-Polarized Butler Matrix for Base Stations With Polarization Diversity. *IEEE Trans. Microw. Theory Tech.* 2018;66(12):5543–5553. 10.1109/TMTT.2018.2880786

[5] Tsokos C : Analysis of a Multibeam Optical Beamforming Network Based on Blass Matrix Architecture. *J. Lightwave Technol.* 2018;36(16):3354–3372. 10.1109/JLT.2018.2841861

[6] Fonseca NJG : Printed S-band 4 × 4 nolen matrix for multiple beam antenna applications. *IEEE Trans. Antennas Propag.* 2009;57(6):1673–1678. 10.1109/TAP.2009.2019919

[7] Ren B Arigong M Zhou JD : A Novel Design of 4 × 4 Butler Matrix With Relatively Flexible Phase Differences. *IEEE Antennas Wirel. Propag. Lett.* 2016;15(c):1277–1280. 10.1109/LAWP.2015.2504719

[8] Wang Y Ma K Jian Z : A low-loss butler matrix using patch element and honeycomb concept on SISL platform. *IEEE Trans. Microw. Theory Tech.* 2018;66(8):3622–3631. 10.1109/TMTT.2018.2845868

[9] Rezvani M Nikmehr S Ghanbari L : A novel Butler matrix feeding system for BST multi-band antennas. *IEICE Electron. Express.* 2013;10(2):1–6. 10.1587/elex.10.20120606

[10] Ibrahim SZ Bialkowski ME : Wideband butler matrix in microstrip-slot technology. *APMC 2009 - Asia Pacific Microw. Conf. 2009.* 2009; pp.2104–2107.

[11] Karimbu Vallappil A Rahim MKA Khawaja BA : Compact Metamaterial Based 4 × 4 Butler Matrix with Improved Bandwidth for 5G Applications. *IEEE Access.* 2020;8:13573–13583. 10.1109/ACCESS.2020.2966125

[12] Hsieh CH Lin YT Jhan HC : Application of a two-dimensional butler matrix antenna array for tile-based beamforming. *IEICE Electron. Express.* 2019;16(11):1–6. 10.1587/elex.16.20190272

[13] Tajik Shafiei Alavijeh A Fakharzadeh M : Asymmetrical 4×4 butler matrix and its application for single layer 8×8 butler matrix. *IEEE Trans. Antennas Propag.* 2019;67(8):5372–5379. 10.1109/TAP.2019.2916695

[14] Shao Q Chen FC Chu QX : Novel Filtering 180° Hybrid Coupler and Its Application to 2 × 4 Filtering Butler Matrix. *IEEE Trans. Microw. Theory Tech.* 2018;66(7):3288–3296. 10.1109/TMTT.2018.2829894

[15] Cao Y Chin KS Che W : A Compact 38 GHz Multibeam Antenna Array with Multifolded Butler Matrix for 5G Applications. *IEEE Antennas Wirel. Propag. Lett.* 2017;16(c):2996–2999. 10.1109/LAWP.2017.2757045

[16] Lian JW Ban YL Xiao C : Compact Substrate-Integrated 4 × 8 Butler Matrix with Sidelobe Suppression for Millimeter-Wave Multibeam Application. *IEEE Antennas Wirel. Propag. Lett.* 2018;17(5):928–932. 10.1109/LAWP.2018.2825367

[17] Rahimi R Shahbazpanahi S : A Two-Way Network Beamforming Approach Based on Total Power Minimization with Symmetric Relay Beamforming Matrices. *IEEE Access.* 2017;5(c):12458–12474. 10.1109/ACCESS.2017.2710908

[18] Djerafi T Fonseca NJG Wu K : Architecture and implementation of planar 4 ×4 Ku-Band nolen matrix using SIW technology. *Proc. 2008 Asia Pacific Microw. Conf. APMC 2008.* 2008; pp.4–7.

[19] Fonseca NJG Ferrando N : Nolen matrix with tapered amplitude law for linear arrays with reduced side lobe level. *Proceedings of the Fourth European Conference on Antennas and Propagation.* 2010; pp.1–5.

[20] Sivasundarapandian Suriyakala CD : A new multi-band multiple beam forming nolen matrix antenna feeding network for cognitive radio. *Int. J. Eng. Technol.* 2014;6(5):2061–2069.

[21] Hussein YM Rahim MKA Murad NA : Low Loss Wideband 4×4 Butler Matrix Networks Based on Substrate Integrated Waveguide for 5G Applications. *IEEE Access.* 2024;12:7896–7910.

[22] Almeshehe MW : Low loss waveguide-based Butler matrix with iris coupling control method for millimeterwave applications. *Waves Random Complex Media.* Feb. 2021;33:372–392. 10.1080/17455030.2021.1880032

[23] Pezhman MM Heidari A-A Ghafoorzadeh-Yazdi A : A novel single layer SIW 6 × 6 beamforming network for 5G applications. *AEU-Int. J. Electron. C.* Oct. 2022;155:154380. 10.1016/j.aeue.2022.154380

[24] Deng J-Y Zhang Y Lin W : Compact Multibeam Antenna Array Facilitated by Miniaturized Slow Wave Substrate Integrated Waveguide Butler Matrix. *IEEE Trans. Antennas Propag.* Jan. 2024;72:9561–9564. 10.1109/TAP.2024.3463203

[25] Idury SK Delmonte N Silvestri L : Design and realization of a compact substrate inte-grated coaxial line butler matrix for beamforming applications. *Int. J. Microw. Wirel. Technol.* Jan. 2024;16(5):812–818. 10.1017/S1759078723001502

[26] Liew CP : Butler Matrix Based on Substrate Integrated Waveguide Without Crossover and Phase Shifter for Millimeter Waves Applications. *Zenodo.* Sept. 2025;8. 10.5281/zenodo.17076167 PMC13264693 42299363

