# Butler Matrix Based on Substrate Integrated Waveguide Without Crossover and Phase Shifter for Millimeter Waves Applications

**DOI:** 10.12688/f1000research.168318.2

**Published:** 2026-04-24

**Authors:** Nawres Nayyef, Kim Geok Tan, Hussam Keriee, Zahriladha Zakaria, Chia Pao Liew, Chockalingam Aravind Vaithilingam

**Affiliations:** 1 Multimedia University Faculty of Engineering and Technology, Ayer Keroh, Malacca, Malaysia; 2 Centre for Advanced Analytics, CoE for Artificial intelligence, Multimedia University, Ayer Keroh, Malaysia; 3 Ministry of Higher Education and Scientific Research, Baghdad, Iraq; 4 Faculty of Electronics and Computer Engineering, Universiti Teknikal Malaysia Melaka, Durian Tunggal, Malacca, Malaysia; 5 School of Energy and Chemical Engineering, Xiamen University Malaysia, Sepang, Selangor, Malaysia; 6 Taylor's University School of Engineering, Subang Jaya, Selangor, Malaysia

**Keywords:** Butler matrix, Hybrid coupler, Vias, Substrate integrated waveguide, 5G

## Abstract

**Background:**

A novel low-loss 4 × 4 Butler Matrix structure for 26 GHz operation is designed in this study. The proposed 4 × 4 antenna Butler matrix comprises an inscribed structure, which makes it fundamentally different from traditional beam-forming networks in 5G 26-GHz applications. This paper presents a compact wideband 4×4 Butler Matrix implemented using Substrate Integrated Waveguide (SIW) technology. In contrast to conventional Butler Matrices that rely on crossovers and discrete phase shifters, the proposed design employs only SIW hybrid couplers to realize the required phase progression and signal distribution. Eliminating crossovers and phase shifters significantly reduces structural discontinuities, junction losses, and frequency-dependent phase errors, thereby enhancing wideband performance. Moreover, the simplified topology minimizes fabrication complexity and improves suitability for planar and monolithic integration, making the proposed matrix well suited for compact millimeter-wave beamforming front ends in 5G and beyond systems.

**Method:**

This novel Butler matrix comprises four hybrid couplers without crossovers or phase shifters. To reduce the size, magnitude, and phase difference, and to increase the bandwidth, interdigital and non-metallic vias are used in the coupler design. The performance of the proposed matrix was simulated using the CST software and implemented on a Rogers 8085 substrate. It was also interfaced with four substrate-integrated waveguide slot antennas to demonstrate its distinct beam scanning capability.

**Results:**

The matrix yielded an error magnitude loss of 1 dB and a phase error of 1.7° with a broad operational bandwidth of 3 GHz. The measured coupling factor of the 4 × 4 Butler matrix at 26 GHz is -6 ±1 dB at the output. The measured results validate that four phase-scanning states at -14.3°, 14.88°, -32.21°, and 31.33° can be realized with return losses of less than -10 dB.

**Conclusions:**

The proposed design achieves a compact, efficient, and low-loss beamforming solution suitable for 5G applications at 26 GHz, demonstrating distinct scanning features without the need for crossovers or phase shifters.

## Introduction

Over the past few years, phased arrays and beamforming have become essential elements in multiple fields, particularly in 5G wireless communication systems. An antenna array is typically controlled by a feeding network that controls the phase shift and amplitude of the respective antenna elements. ^1^ The antenna array is most easily articulated in these embodiments because the radiation beam is steered by the antenna array towards the selected position. This feature gives rise to three different types of feed networks for the control of antenna arrays: parallel-feed networks, series-feed networks, and matrix-based networks. ^2^ Wide-tuning phase shifters are required for both parallel and series feed networks, and they restrict the capability of the antenna array to simultaneously form more than one beam at the same time. However, this was not the case for matrix feed networks, which had hybrid couplers, crossover, and a phase shifter network. ^3^ In addition, matrix networks have the special property that multiple beams can be produced simultaneously. Different matrix feeding networks have been designed, such as the widely used Butler, Blass, and Nolen matrices. ^4– 6^ The Butler matrix is well known for its symmetric design with an equal number of inputs and outputs. Equal-magnitude signals with progressive phase differences are emitted from the output ports ^7^ of each input port, and the simultaneous radiation of the two beams can be accomplished. The Butler matrix has been widely investigated for its low loss, ^8^ multiband, ^9^ wideband, ^10^ small size, ^11^ and millimeter-wave operating ranges. ^12^

Various integrated platforms have been explored for the implementation of switching and beamforming matrices, including microstrip, coplanar waveguide (CPW), coaxial, and photonic technologies. Each platform offers distinct advantages, but also exhibits inherent limitations when applied to wideband millimeter-wave matrix architectures.

Microstrip-based switching matrices are widely reported due to their low cost, planar form factor, and ease of integration with active components. ^13^ However, Ref. 14 at millimeter-wave frequencies, microstrip implementations suffer from increased radiation losses, surface-wave excitation, and dispersion effects, which limit achievable bandwidth and degrade phase accuracy in large matrix configurations. Coplanar waveguide (CPW) ^15^ platforms alleviate some integration challenges and provide improved grounding flexibility, yet they remain susceptible to radiation leakage and conductor loss at high frequencies, particularly in dense multi-port networks ^16^ Coaxial and waveguide-based matrices offer superior isolation and low loss, making them attractive for high-performance systems. Nevertheless, their bulky three-dimensional structures, limited scalability, and high fabrication and assembly costs restrict their suitability for compact and highly integrated 5G and beyond applications. More recently, photonic switching matrices ^17^ have been proposed to enable ultra-wide bandwidth and immunity to electromagnetic interference. Despite these advantages, photonic solutions typically require complex electro-optic conversion stages, precise alignment, and specialized fabrication processes, resulting in increased system complexity and cost.

In comparison, Substrate Integrated Waveguide (SIW) ^18^ technology combines the low-loss and high-isolation characteristics of conventional waveguides with the planar manufacturability of printed circuit technologies. This hybrid nature makes SIW particularly well suited for integrated wideband switching matrices, offering reduced radiation loss, improved phase stability, and excellent scalability while maintaining compatibility with standard PCB fabrication. Consequently, the SIW-based Butler Matrix proposed in this work provides a balanced solution that directly addresses the limitations of alternative integrated platforms.

Furthermore, studies on symmetric structures at two scales, including the 4 × 4 ^19^ and 8 × 8 networks, have also been performed. ^20^ Later studies employed the Butler matrix for non-symmetric structures, such as the 2 × 4 network, ^21^ 3 × 4 network, ^22^ and 4 × 8 network. ^23^ The Blass matrix is a cross-over free full-rank matrix with half as many input and output ports as the Butler matrix, but load terminations in the Blass matrix substitute for the crossovers of the Butler matrix. There are structural limitations of the Blass matrix that could lead to partial signal flow towards the termination loads, which causes the efficiency to be less than that of the Butler matrix. ^5^ To overcome this drawback, improvements in the Butler matrix have been suggested, focusing on a reduction in the number of components and element footprints. However, for lower 5G bands such as 26 GHz, beamforming components (e.g., couplers, phase shifters, and antenna elements) have limited flexibility and high signal loss. ^24^ An early beamforming network was introduced in Ref. 25 where it incorporated a Butler matrix algorithm in combination with a 90° coupler. Another development in beamforming networks was considered in Ref. 26, which reports on a series-fed matrix design that suppresses sidelobes at 28 GHz. In Ref. 27, a microstrip beamforming system was presented for multiband operations at 26, 28, and 30 GHz. The coupler used in this configuration was H-type with a short slot. One recent example is in Ref. 28, where the work is at 28 GHz with a 4 × 4 Butler matrix structure for a 2-D beamforming network that uses three couplers and three phase shifters. Another example of a compact form factor is the 4 × 4 beamforming network at 28 GHz, which was designed in Ref. 29 using lumped element couplers to alleviate the matrix size. In addition, a 4 × 4 beamforming network using 90-degree coupler was presented in Refs. 30 and 31 for 28 GHz, with four Substrate Integrated Waveguide (SIW) couplers, one crossover, and two phase shifters. Unfortunately, this structure is large in size with a very high IL and phase difference, which consequently results in size and phase error difficulties in these prior realizations at such increased 5G frequencies.

Beamforming networks and switching matrices are fundamental components in modern multi-antenna systems, enabling signal routing, phase control, and spatial beam steering in applications such as 5G millimeter-wave communications, radar, and satellite systems. Within this broad class, ^32^ integrated switching matrices can be generally categorized into **narrowband** and **wideband** architectures, depending on their operational bandwidth, phase stability, and amplitude balance characteristics.

Narrowband matrix architectures, including conventional waveguide and microstrip Butler matrices, are typically optimized for operation around a single center frequency. While these solutions offer compact layouts and relatively low insertion loss, ^33^ their performance rapidly degrades outside the design band due to frequency-dependent phase errors and impedance mismatch. Such limitations restrict their suitability for emerging wideband and multi-carrier millimeter-wave systems. In contrast, wideband matrix architectures employ wideband couplers, phase shifters, or hybrid implementations to maintain stable phase progression and amplitude balance over an extended frequency range. These designs are particularly relevant for 5G and beyond systems, where wide instantaneous bandwidth, beam squint mitigation, and integration compatibility are critical design requirements. However, many reported wideband matrices rely on multilayer structures, complex transitions, or mixed technologies, which increase fabrication complexity and cost.

In this context, Substrate Integrated Waveguide (SIW) technology provides an attractive platform for realizing wideband integrated switching matrices by combining the low-loss characteristics of conventional waveguides with the planar manufacturability of printed circuits. The proposed SIW Butler Matrix in this work is positioned within the **wideband matrix architecture class**, specifically targeting wideband operation with stable phase differences and reduced insertion loss while maintaining a fully planar and fabrication-friendly structure. By employing only SIW hybrid couplers without additional phase-shifting elements, the proposed design offers a simplified topology that directly addresses the bandwidth and integration challenges encountered in existing matrix implementations.

In this paper, a novel 4 × 4 Butler matrix with low loss, compact size, small amplitude, and phase error across the band is presented, based on a normal and unequal separation vector structure along the waveguide walls. The remainder of this paper is organized as follows. First, the topology of the design and equations of the Butler matrix and its variants, as well as those of the design of couplers, are studied. Second, a comprehensive 4 × 4 Butler matrix approach is presented with flexible performance. Subsequently, the measurement results of the proposed 4 × 4 Butler matrix are systematically compared and discussed with respect to simulations and some works presented in the literature. Finally, the major findings are summarized. Taken together, this novel Butler matrix structure is promising for improving beamforming networks of 5G wireless communication systems.

## Method – Design of beamforming

**Figure 1.**
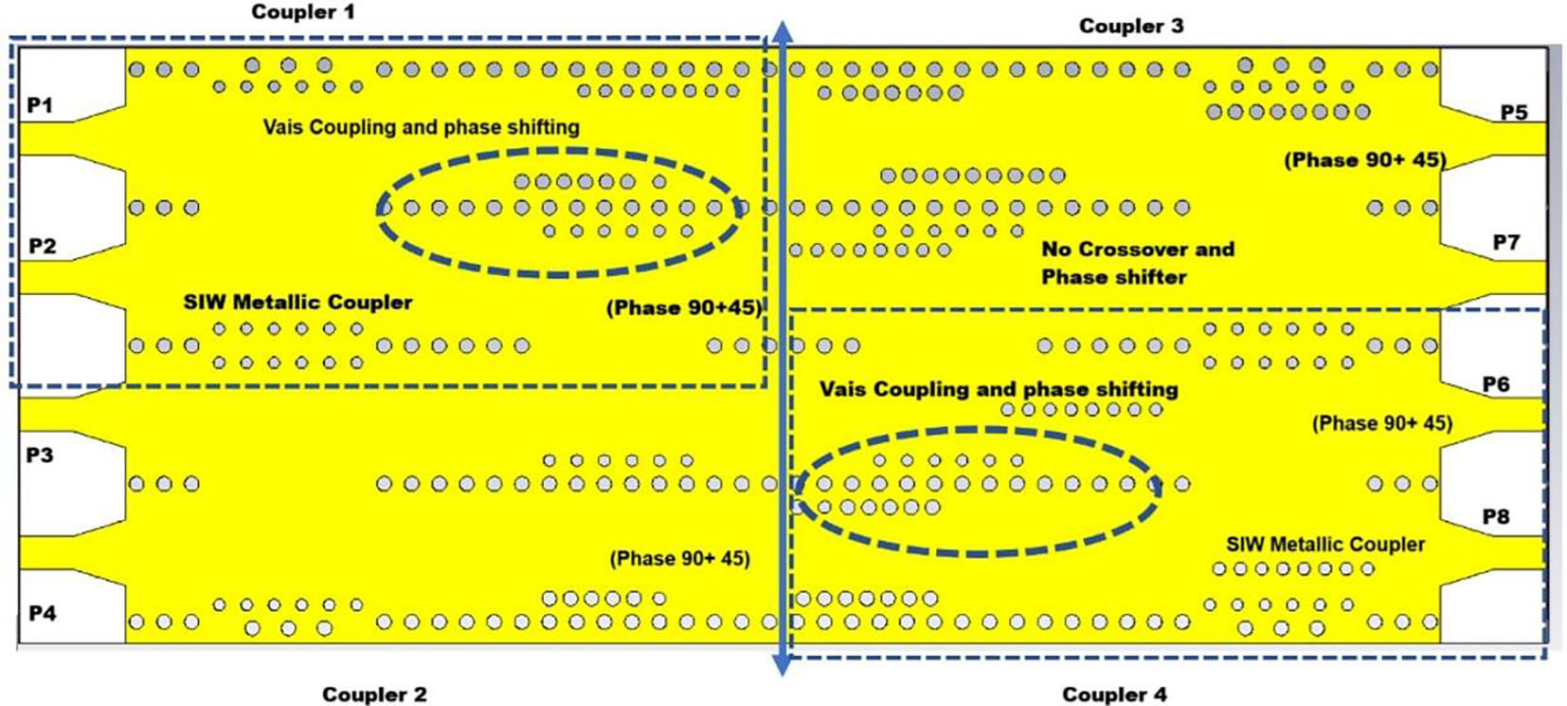
The structure of 4 × 4 SIW Butler Matrix.

Figure 1 presents the structure of the 4 × 4 Butler matrix, where the four input ports are defined as (P1, P2, P3, and P4) and the four output ports are represented as (P5, P6, P7, and P8). The Butler matrix presented in Figure 1 contains only four hybrid couplers. All couplers have a single coupling constant that is distributed along the matrix equally between the lower and upper parts. To maintain edge-to-edge uniformity of signal levels (for the output of Butler matrix) and signal integrity of lines, the coupling ratios of (C1) and (C2) were selected. Hence, the coupling ratio of the couplers at the Butler matrix outputs can be expressed as Ref. 28.

**Figure 2.**
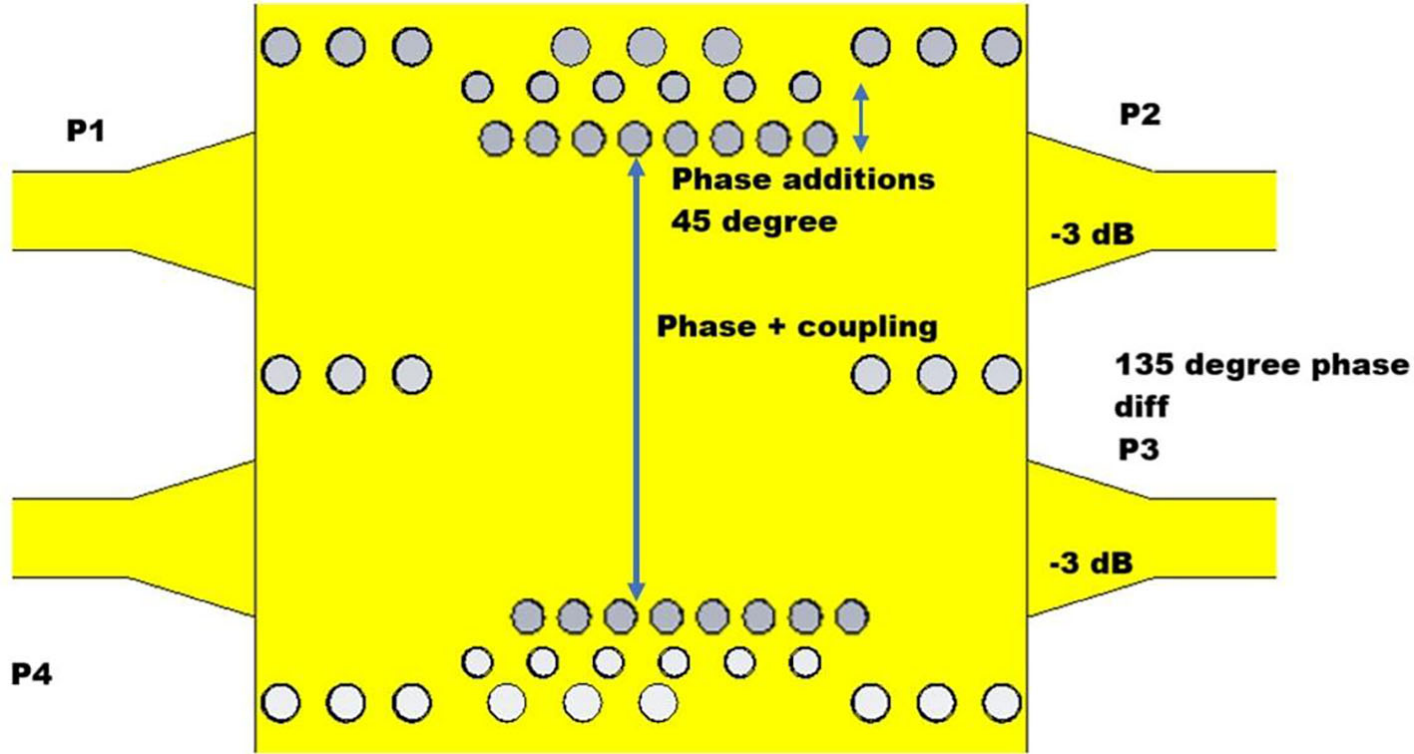
The structure of SIW Hybrid coupler with phase difference of 135 degree.

The schematic structure of the two-section VAI hybrid coupler used for the speed demonstration with a center frequency of 26 GHz is illustrated in Figure 2. The coupler is designed to have a symmetric four-port structure (P1–P4) and is based on a via-based internal coupler structure for coupling and phase modulation. Key findings included:

Bandwidth:

$$\mathrm{FBW}=\frac{f_{H-}f_{L}}{f_{0}}$$ (1)

where $f_{H}$ and $f_{L}$ are the upper and lower frequency limits of the passband and $f_{0}\;$ the center frequency. A fractional bandwidth (FBW) of 55.42% (ranging from 24 to 27 GHz),

The proposed power splitter is constructed using substrate-integrated waveguide (SIW) technology, the two power-dividing arms of which are synthesized by electromagnetic coupling and phase manipulations to achieve the desired characteristics. The design embodies 50 Ω quarter-wavelength transmission lines as the basic structure of the coupler arms. The arms are realized by a set of carefully optimized strip-line widths based on the microwave design equations found in Refs. 30 and 31. Rows, which act as artificial magnetic conductors, are mainly utilized to confine electromagnetic fields but also affect impedance matching and phase response. This is evident in Figure 2, which also shows the signal flow from port to port. The input power at port P1 is equally divided into ports P2 and P3, with-3 dB at each port. In addition, the coupler introduces a well-defined phase relationship, which provides 45° and constructive phase coupling at the central transmission axis, whereas the phase shift difference between the output ports is 135°. This phase shift is essential for many beamforming systems, such as the Butler matrix in 5G phased arrays. To widen the operating band, an arm-shaped structure was used for each coupling section. The modified arms act as impedance transformers to make the required impedance transitions (Z _1_, Z _2_, Z _3_) over the band. The design equations are formulated in closed form as related to even- and odd-mode analyses to obtain low reflection and high isolation between ports. In general, the design exhibits good engineering accuracy, particularly with respect to impedance tuning, phase control, and coupling balance its due regard. These hybrid couplers are important in millimeter-wave transceivers, where the architecture, efficiency, and bandwidth require compactness, and wideband performance is achieved by providing degrees of freedom for phase control and power division in the physical layout. When a single is applied to port P1, the power is divided equally between ports P2 and P3, with a loss of approximately 3 dB. The signal path includes phase-adding means: in the vertical direction, it is a 45° phase adder, while the output ports P2 and P3 retain their 135° phase difference, which is essential for modern millimeter-wave beamforming systems where, for example, the Butler matrix is implemented. In the revised crossover-free, phase-shifter-free topology, the required phase distribution is realized through the intrinsic phase properties of the SIW hybrid couplers and path-length differences in the interconnecting SIW transmission sections.

For an ideal 3-dB quadrature hybrid coupler:

• The two output signals have equal magnitude.
• The relative phase difference between the “through” and “coupled” ports is approximately ±90° (sign depends on port definition).

Thus, the cumulative phase at each output port can be written as a sum of:

1. hybrid-coupler phase terms, and
2. propagation phase terms along SIW paths.

$$\varphi_{\text{out},n}=\sum_{i=1}^{N_{c}}\text{φhyb},i+\;\sum_{m=1}^{N_{p}}\beta_{g}L_{m}$$ (2)

Where:

•
$N_{c}$ = number of couplers on the signal route
•
$N_{p}$ = number of SIW path segments
•
$L_{m}$ = physical length of the m-th segment
•
$\beta_{g}$ = guided propagation constant of SIW
  For SIW, the guided wavenumber can be approximated by:

$$\beta_{g}=\sqrt{k_{0}{}^{²}\varepsilon_{r}-{\left(\frac{\pi }{a_{\mathrm{eq}}}\right)}^{²}}$$ (3)

where $k_{0}=\frac{2\pi }{\lambda_{0}}$

*ε*_*r*_ is the substrate relative permittivity, and $a_{\mathrm{eq}}$ is the equivalent SIW width determined from the via diameter and pitch. This expression allows computing the expected phase shift of any interconnect line:

$$\varphi_{\text{prop}}=\beta_{g}L$$ (4)

**Figure 3.**
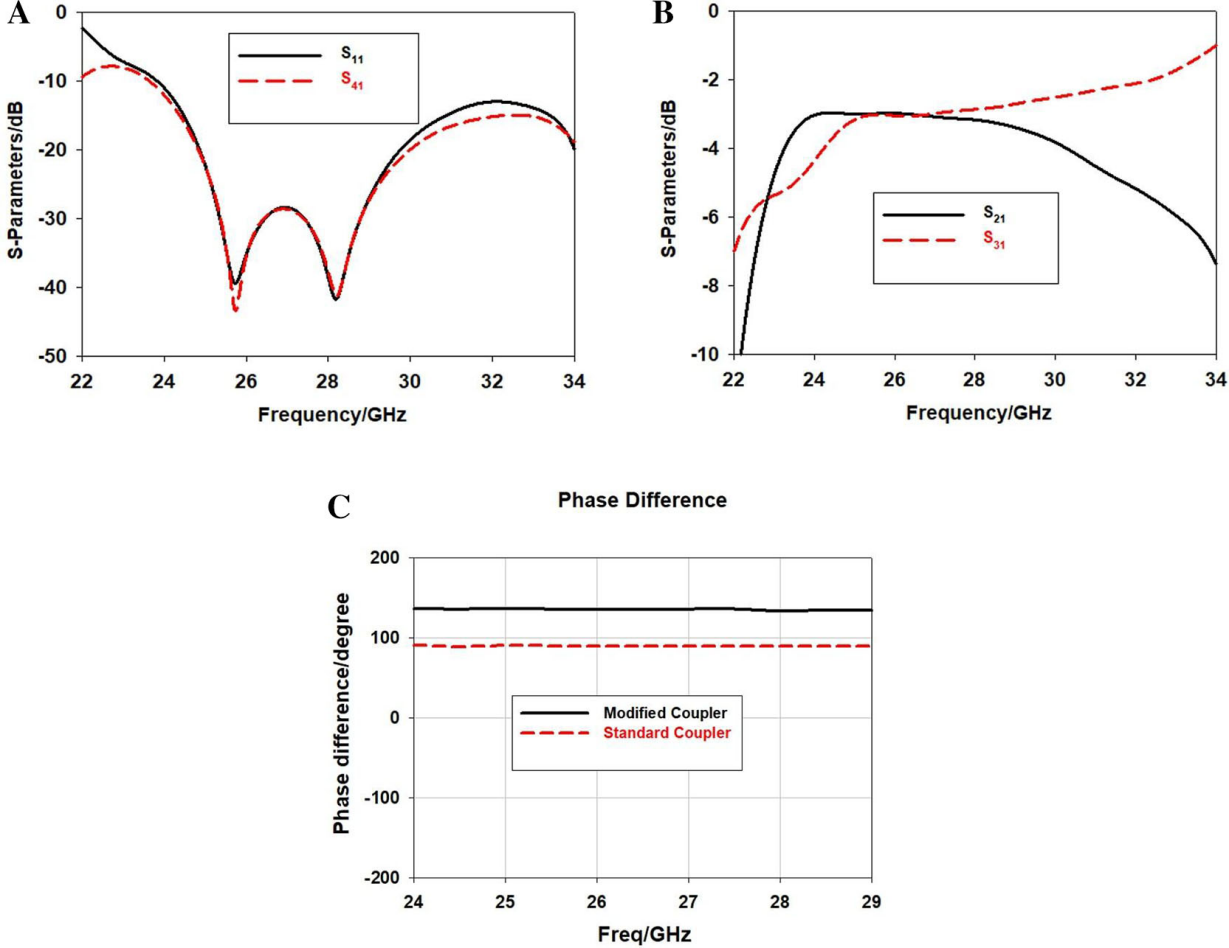
Simulated response for designed hybrid coupler. (A) Return loss and isolation, (B) Output power, (C) Phase difference vs standard coupler.

The response of the coupler was characterized using numerical simulations based on full-wave electromagnetic equations. The obtained S-parameters indicate the operational bandwidth, matching properties, and isolation performance of the coupler and are depicted in Figure 3. As shown in Figure 3(A), a sharp dip below -30 dB in | *S*_11_| was observed at a center frequency of 26 GHz, indicating a good impedance-matching Doppler radar. The isolation from port P1 to isolated port P4 ( $S_{41}$ ) follows a similar behavior, achieving values less than -30 dB at resonance and a minimum close to -40 dB. These findings verify the effective suppression of unwanted reflections and high isolation between noncoupled ports, which is an essential figure of merit for hybrid couplers. The coupling levels from input P1 to output ports P2 ( $S_{21}$ ) and P3 ( $S_{31}$ ) are well suppressed, as shown in Figure 3(B). At 26 GHz, both $S_{21}$ and $S_{31}$ meet at about -3.1 dB, confirming the desired equal power splitting. Furthermore, making a power splitter at a power splitting range of -3 dB to -4.5 dB over a 5-GHz frequency range (from 24.5 to 29.5 GHz), that is, operation bandwidth is also realized. As shown in Figure 3(C), the phase difference between ports P2 and P3 was 135°, not the standard 90°, which reflects a deliberate design choice. This enhancement of the phase difference enables the coupler to integrate both the coupling and phase-shifting functions into a single device, thereby combining the roles of a hybrid coupler and phase shifter.

**Figure 4.**
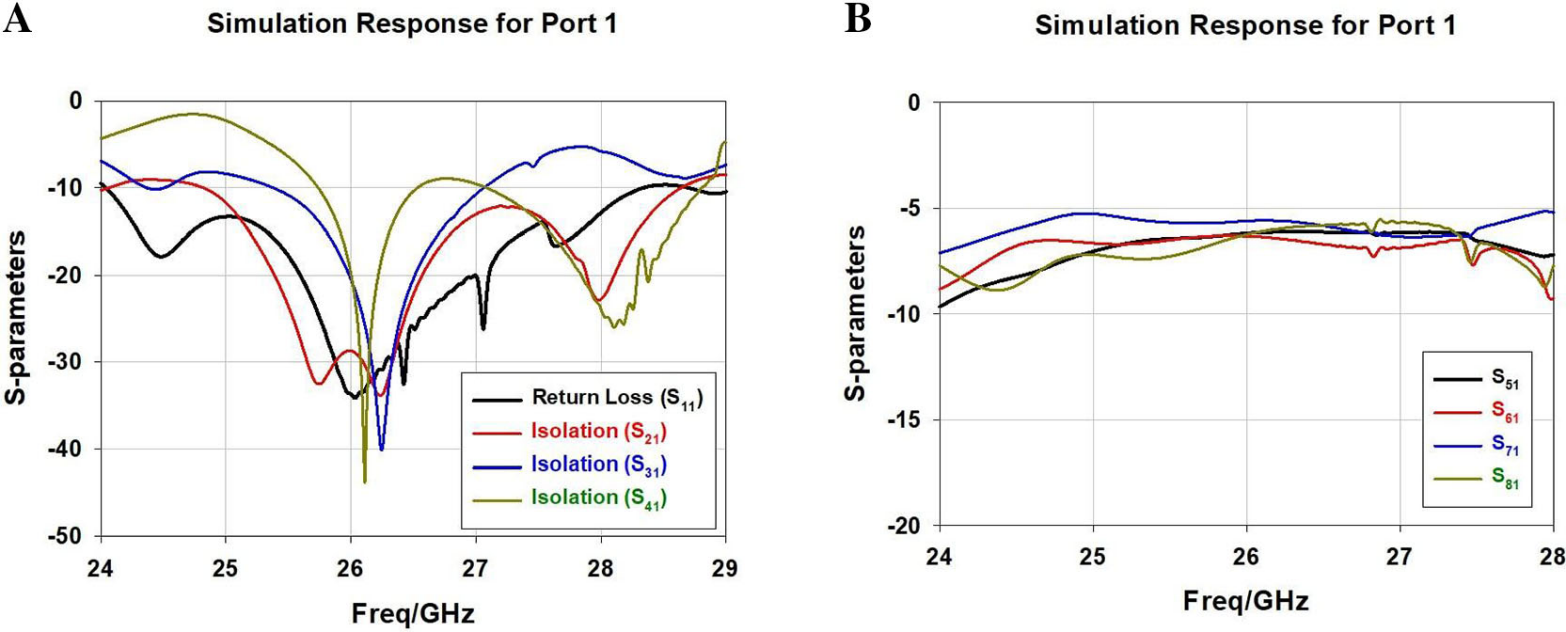
Simulated Port 1 of BM. (A) Return loss and isolated ports, (B) Output ports.

**Figure 5.**
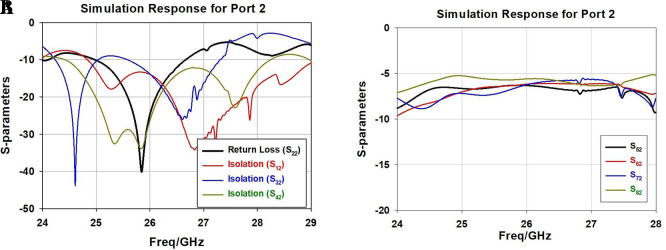
Simulated Port 2 of BM. (A) Return loss and isolated ports, (B) Output ports.

**Figure 6.**
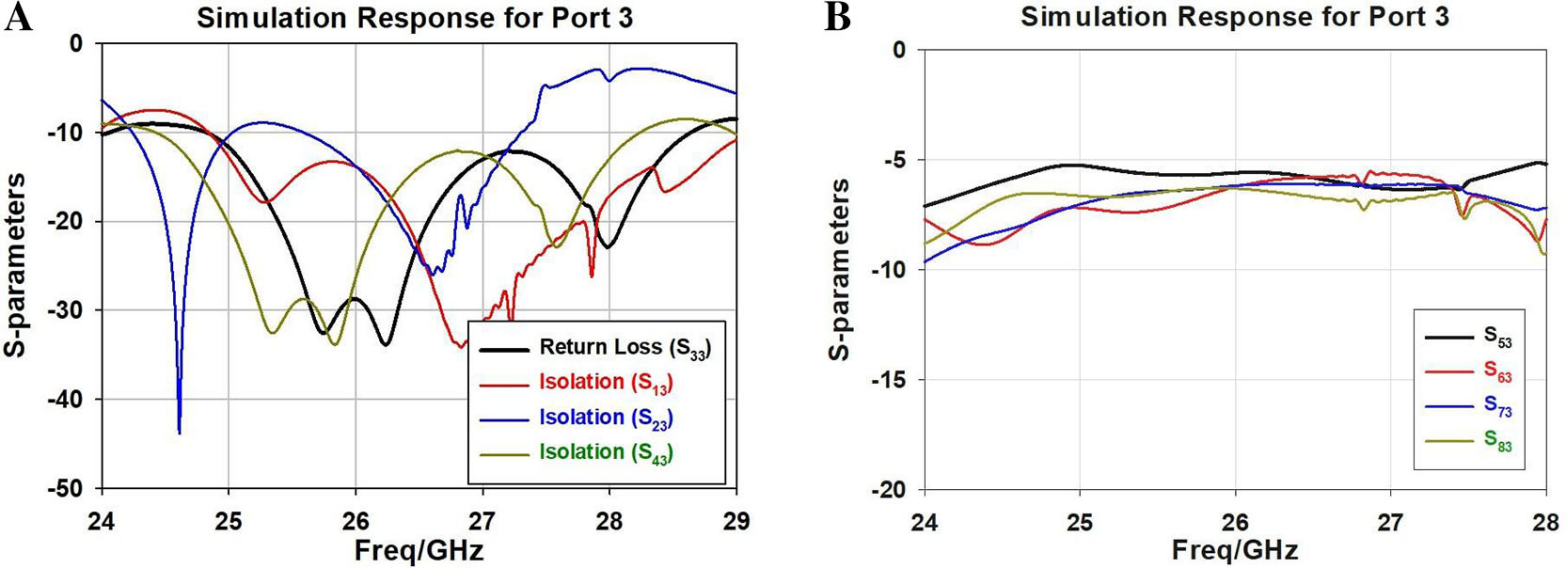
Simulated Port 3 of BM. (A) Return loss and isolated ports, (B) Output ports.

**Figure 7.**
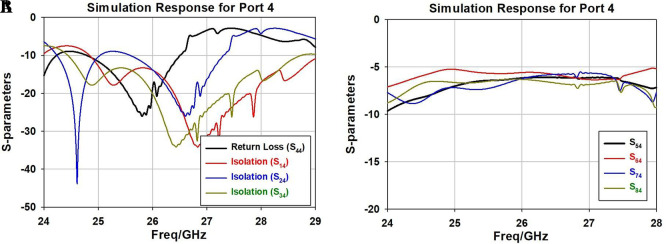
Simulated Port 4 of BM. (A) Return loss and isolated ports, (B) Output ports.

As mentioned earlier, Figure 1 shows the implementation of a 4 × 4 Butler matrix design and the assembly of four couplers. A simulation using Computer Simulation Technology (CST) was carried out to prove the feasibility of the 4 × 4 Butler matrix. The S-parameters are linked to the return loss, isolation, and transmission coefficients, as shown in Figure 4(A), 5(A), 6(A), and 7(A), respectively. As shown in Figure 4, the return loss of input port 1 shows acceptable results in all bands and is below -10 Db. More precisely, at 26 GHz, the measured return loss values were *S*_11_ = −20:4 dB, *S*_21_ = −18:7 dB, and *S*_31_ = −22:9 dB, yielding a fractional bandwidth of 60% over all input ports. This demonstrates that good isolation was achieved for all the ports. Figure 4(B) also shows the transmission coefficients when port 1 is powered on, it produces S _51_ = -5.8 dB, S _61_ = -6.12 dB, S _71_ = -6.12 dB > S _61_, and S _81_ = -6.5 dB. These results indicate that the power is distributed equally in the outputs if Port 1 is excited. Thus, the calculated S-parameter results corresponded to the design specifications of the 4 × 4 Butler matrix. The input return loss at input port 2 is superior and less than -10 dB. At 26 GHz, for example, the return loss measurements read *S*_22_ = -20.4 dB, *S*_12_ = -18.7 dB and *S*_13_ = -22.9 dB, which encompasses a fractional bandwidth of 60% for all of the input ports. This shows that the isolation between the ports is good. Figure also shows the behaviour of the transmission coefficients when Port 2 is on. As shown in Figure 5(B) The measured values are *S*_52_ =-6.8 dB, *S*_62_ = -6.35 dB, *S*_72_= -6.54 dB, and *S*_82_ = -6.55 dB. This implies that equal power is distributed to the outputs with excitation at port 2. The return loss of input port 3 is shown in Figure 6(A), with good performance below -10 dB. Return losses values of *S*_33_ = -25.5 dB, *S*_23_ = -20.7 dB, and *S*_43_ = -24.7 dB at 26 GHz and a fractional bandwidth of 60% are obtained over all input ports This indicates that proper isolation was achieved at each port. The transmission coefficients upon the activation of port 3 are shown in Figure 6(B). The measured values are *S*_53_ = 6.2 dB, *S*_63_ = 5.85 dB, *S*_73_ = 6.22 dB and *S*_83_ = 6.75 dB which implies that power equally distributes among the ports when port 3 is excited. Figure 7 shows the return loss behaviour at input port 4, where excellent performance is observed with a value of less than -10 dB. Remarkably, at 26 GHz, the return loss measurements are *S*_44_ = -24.7 dB, *S*_43_ = -22.8 dB, and *S*_42_ =- 22.6 dB, respectively, which again constitute a 60% fractional bandwidth for all input ports. This means that strong isolation is reached at each port. The transmission coefficient responses upon the activation of port 4 are shown in Figure 7(B). The measured *S*_53_ = -6.8 dB, *S*_63_= -5.47 dB, *S*_73_ = -6.65 dB, and *S*_83_ = -6.52 dB, suggest that when port 4 is involved, the output power is evenly divided.

**Figure 8.**
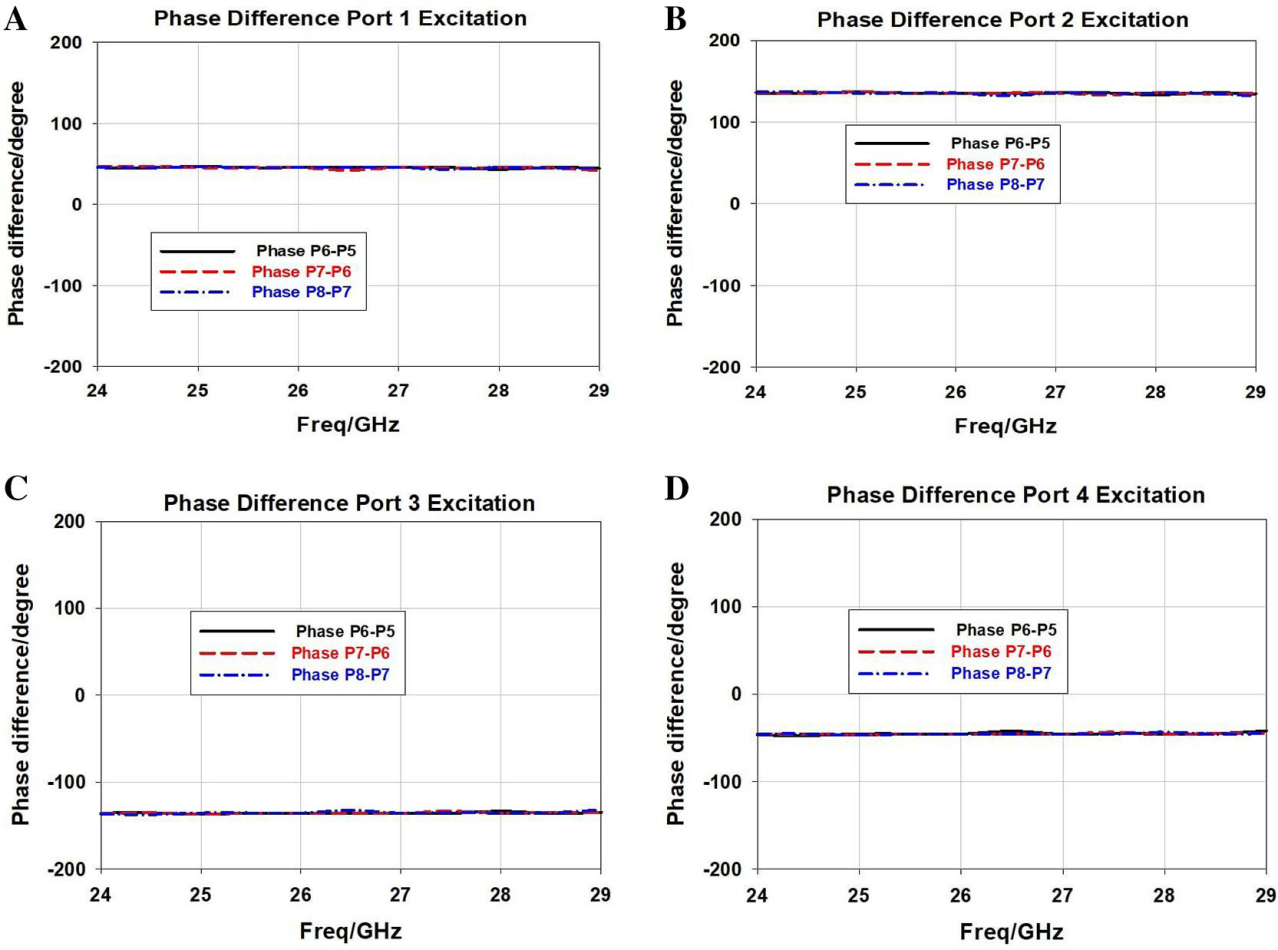
Simulated phase differences of proposed BM. (A) Port 1, (B) Port 2, (C) Port 3, (D) Port 4.

The simulated phase shifts of the output ports with excitation from Port 1 are shown in Figure 8(A). In particular, when port 1 is actuated, the phase dispersion of the outputs at ports 5, 6, 7, and 8 is 45.6°. By turning on port 2, a phase difference of 133.6° can be observed, as shown in Figure 8(B). Note from the accompanying figure that all outputs (1, 2, and 3) have the correct phase of excitation when the signal from port 2 is set. The simulated phase shifts for the output ports under excitation at port 3 are shown in Figure 8(C) together with the phase differences for the four output ports of the 4 × 4 Butler matrix. It can also be found that when port 3 is excited, the simulated phase difference between the outputs (i.e., ports 5, 6, 7, and 8) is -133.23°, as shown in Figure 8(C). -45.6° is the phase difference when port 4 is excited, as shown in Figure 8(D).

## Results and Discussion

**Figure 9.**
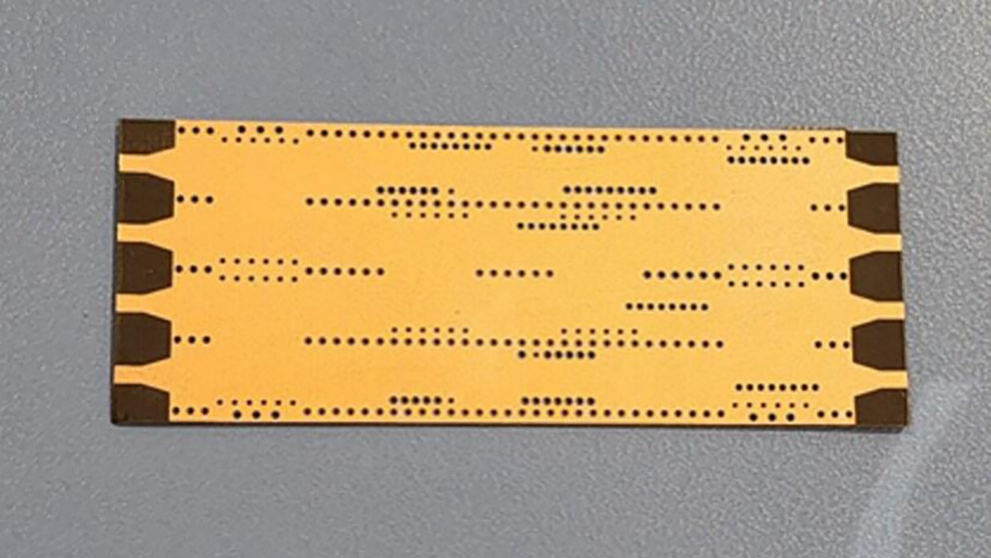
Fabricated 4 × 4 SIW BM.

**Figure 10.**
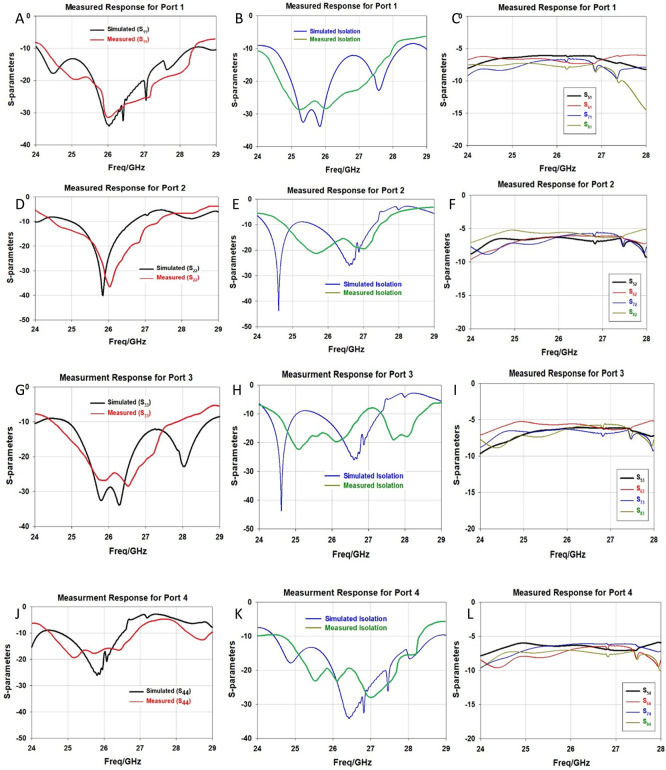
Measured vs. simulated results of SIW BM. (A)(B)(C) Port 1, (D)(E)(F) Port 2, (G)(H)(I) Port 3, and (J)(K)(L) Port 4.

The suggested 4 × 4 Butler matrix design was realized on a Roger substrate with a thickness of *h* = 0.508 mm and relative permittivity of *ε*_r_ = 2.2. Figure 9 shows the manufactured 4 × 4 Butler matrix. The S-parameter tests were performed using a Keysight (Agilent Technologies) field forecast vector network analyzer (VNA) (N9925A), two cables, and six referent dummy loads. The efficiency of the 4 × 4 Butler matrix hardware is demonstrated by its *S*-parameters, as seen in the simulation results shown in Figure 10.

The measured return loss and isolation from the port 1 excitation are plotted in Figure 10(A). The measured *S*_11_ is −31.1 dB at 26 GHz, and the wideband response has a 2 GHz percentage of 60 FBW of operation. The results of these simulations were compared with the measurements. Furthermore, the isolation of ports 2, 3, and 4 at all reconfigurable states is satisfactory and remains less than -10 dB over the desired band, as illustrated in Figure 10(B). The measured transmission coefficients in Figure 10(C) are *S*_51_ = −6.5 dB, *S*_61_ = −6.69 dB, *S*_71_ = −6.79 dB, *S*_81_ = −7.74 dB, which shows an amplitude loss of −1.8 dB. Therefore, a low insertion loss was obtained for the port 1 excitation. The return loss and isolation of the port 2 excitation are shown in Figure 10 (D). In particular, *S*_22_ is measured at -30.3 dB and 26 GHz with a wideband of 2.4 GHz to maintain a 60% FBW, and is set for comparison with the simulated results. On the other hand, the remaining isolations (ports 1, 3, and 4) also perform well, with isolations in the range of −10 dB over the desired frequency band. The transmission coefficients of Figure 10(F) are given as *S*_52_ = -7.5 dB, *S*_62_ = -7.69 dB, *S*_72_ = -6.49 dB, and *S*_82_ = -6.2 dB, indicating amplitude attenuation of -1.5 db. Hence, for the port 2 drive, a low insertion loss is found. The simulated return loss and isolation at the port 3 excitation are shown in Figure 10(G). The measured −25.6 dB *S*_33_ at 26 GHz, together with the wideband characteristic (26 GHz, 55% FBW), is compared with the simulated results. The measured isolations from ports 1, 2, and 4 were well maintained at less than even -10 dB over the designed bandwidth, as depicted in Figure 10(H). The measured transmission coefficients in Figure 10(I) are *S*_53_= -6.58 dB, *S*_63_ = -5.53 dB, *S*_73_ = -7.49 dB and *S*_83_ = -7.3 dB with magnitude loss of –1.6 dB. Accordingly, in the port 3 excitation, a low insertion loss was attained. The measured return loss and isolation for the port 4 excitation are shown in Figure 10(J). This measured *S*_44_= -16.7 dB at 26 GHz over a wideband characteristic of 4 GHz (60% FBW) is then compared to simulated results. In contrast, the measured isolation from ports 1, 2, and 3 exhibits a good performance of lower than -10 dB over the desired bandwidth, as illustrated in Figure 10(D). From the Figure 10(I), the measured transmission coefficients are -7.58 dB ( *S*_54_), -6.87 dB ( *S*_64_), -6.77 dB ( *S*_74_) and –7.58 dB ( *S*_84_), with a magnitude loss of -1.85 dB. Thus, the insertion loss at the port 4 excitation was also low.

**Figure 11.**
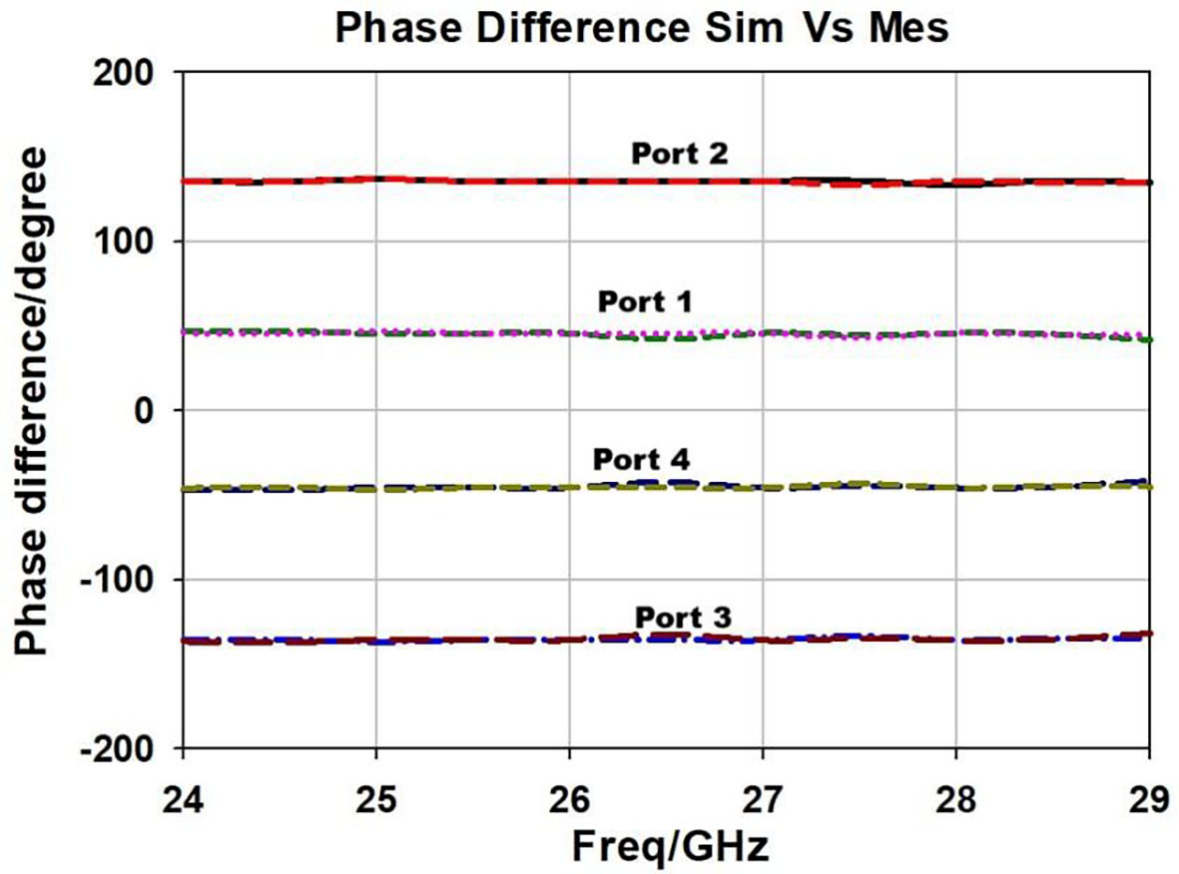
Measured vs simulated phase difference at 26 GHz.

The measured phase difference of the outputs when all the input ports are active is shown in Figure 11. When compared with the simulated phase differences, it was observed that the measured phase difference at the port 1 excitation was 131° with a phase error equal to 1°. When the excitation was performed from ports 2 and 3, the phase differences were 46.05° and -46.04° with phase errors of 1.05° and -1.04° respectively. The measured phase difference at port 4 was − -134° with a phase error of 2°. Therefore, the proposed Butler matrix has a tunable phase difference at the output ports, with an average phase error of 2° at the low-frequency stage.

**Figure 12.**
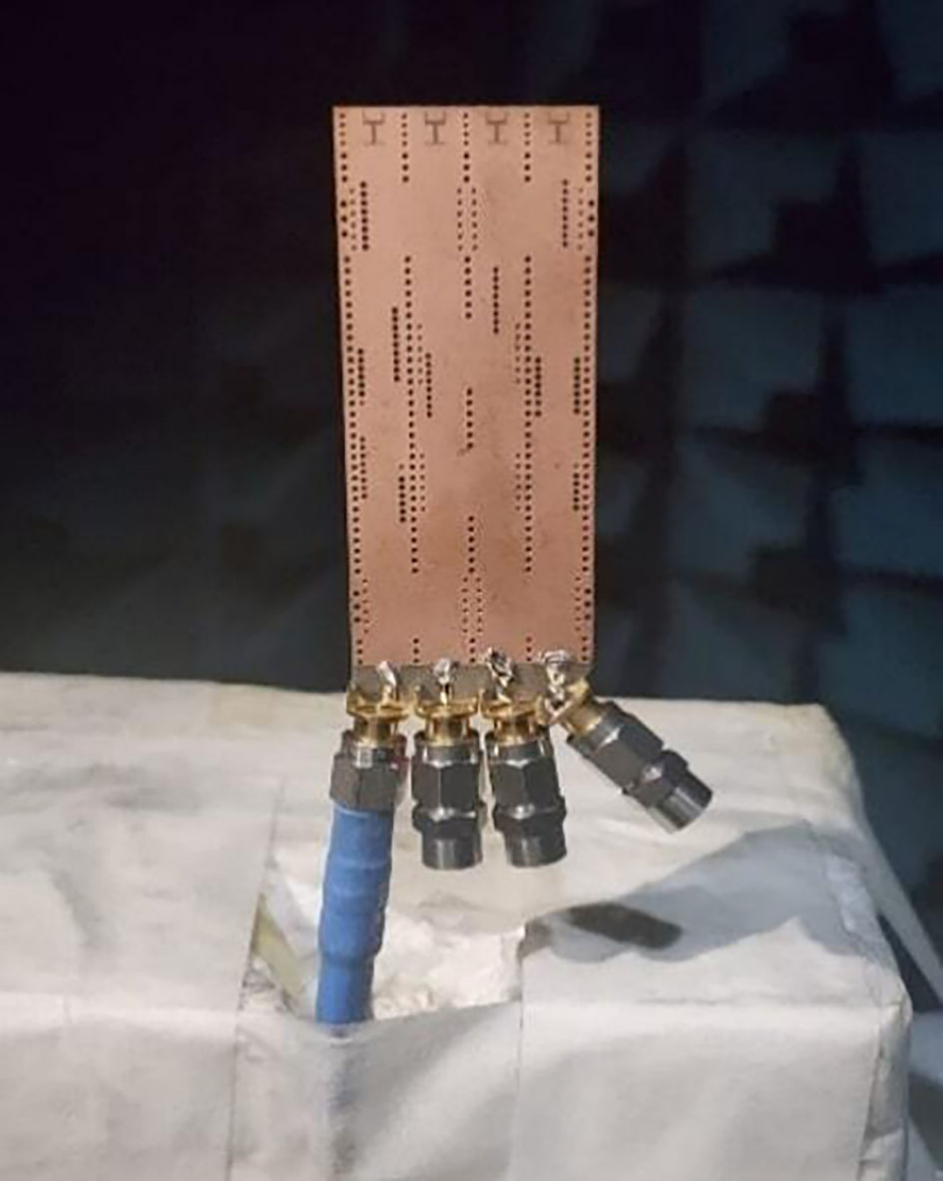
The fabricated beamforming network.

**Figure 13.**
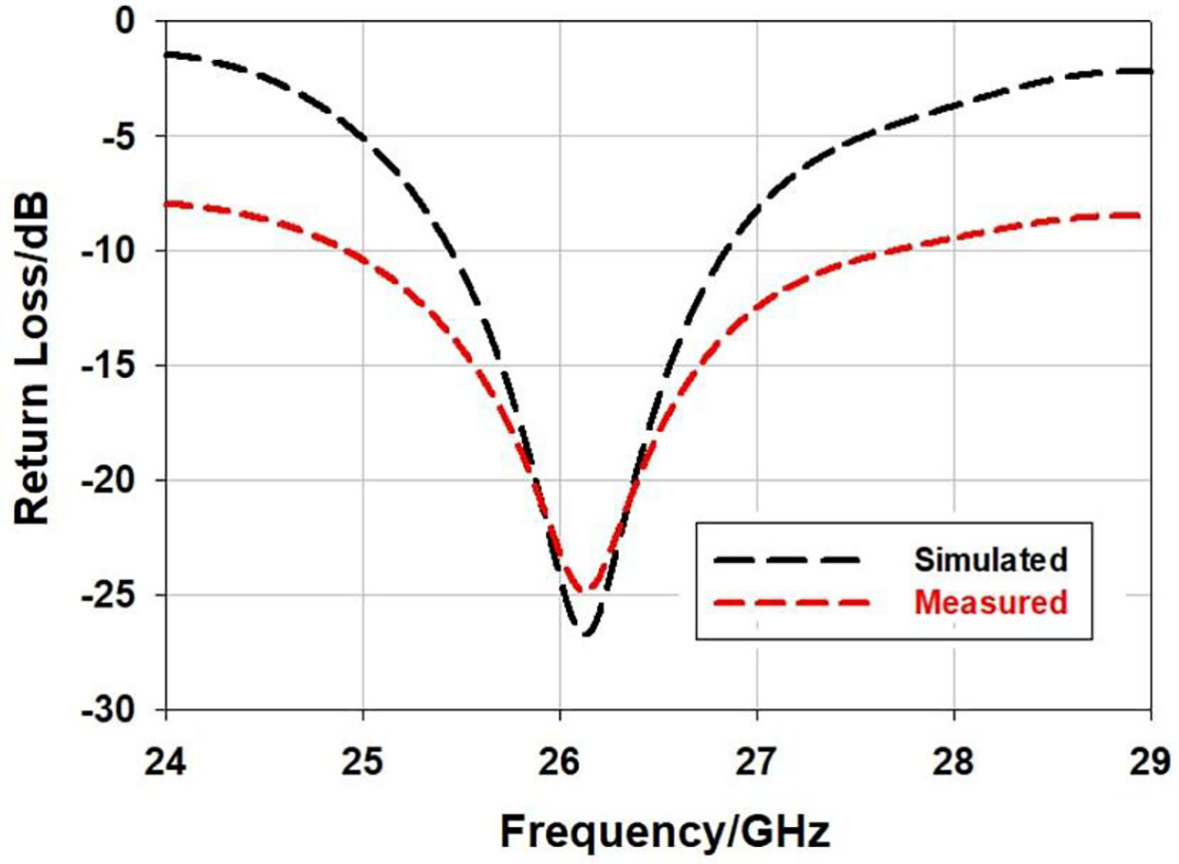
The measured vs simulated return loss of beamforming.

**Figure 14.**
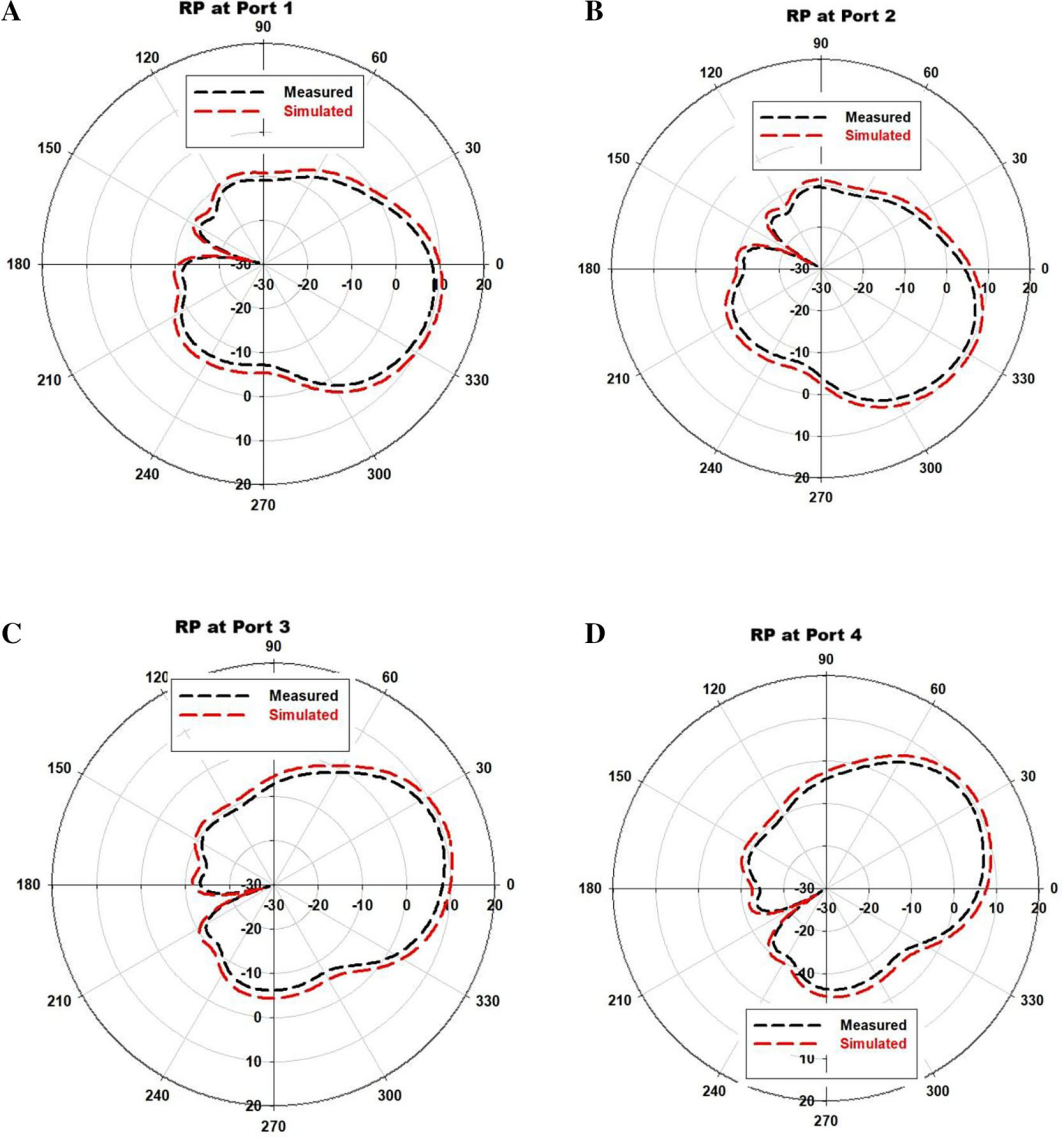
The measured vs simulated RP at 26 GHz. (A) Port 1, (B) Port 2, (C) Port 3, (D) Port 4.

The prototype of the proposed antenna beamforming network with a Butler matrix network is shown in Figure 12. The simulated return loss of the proposed beamforming network is illustrated in Figure 13. At 26 GHz, the return loss for all excited ports is < −10 dB. The operational bandwidth of the proposed Butler Matrix is relatively limited compared to some reported SIW-based implementations. This behavior is primarily attributed to the **cascaded hybrid-coupler topology** employed to eliminate crossovers and discrete phase shifters. In such configurations, the overall bandwidth is governed by the **intersection of the passbands of the individual couplers**, resulting in a reduced effective bandwidth as the number of cascaded stages increases. Additionally, the bandwidth is influenced by the **phase balance requirement** intrinsic to Butler matrices. Even when acceptable impedance matching is maintained over a wider frequency range, stringent phase-error constraints impose a narrower usable bandwidth for accurate beamforming operation. This suggests that the beamforming feeding network performed well at the desired bandwidth. Nonetheless, at ports 2 and 3, a decay in the signal was detected because of the mismatch between the connectors and channels, as well as the losses of the cable. The bandwidth was approximately 2.8 GHz, which suggests that the performance of the beamformer is highly resonant at various frequencies. The measured and simulated radiation patterns for the designed BM in the SIW are shown in Figures 14(A), 14(B), 14(C) and 14(D). Owing to ±31.23° for all ports, 9 dB beams were obtained at ±14.43° and ±11 dB at all ports. Key contribution of this work lies in the **original structural simplicity** and performance advantages of the proposed SIW Butler Matrix compared to existing 4×4 matrix designs that rely on crossovers and discrete phase-shifter networks. Traditional implementations of 4×4 Butler Matrices in microstrip and CPW technologies often incorporate crossovers and fixed electrical-length phase shifters to achieve the required interconnections and phase progression. While functional, these elements introduce additional **insertion loss**, **phase distortion**, and **size penalties**, particularly at millimeter-wave frequencies, due to increased discontinuities, radiation effects, and dispersion.

By contrast, the proposed SIW design employs only SIW hybrid couplers without separate crossovers or discrete phase shifters, leading to a reduction in cumulative junction loss and improved phase fidelity over a wide frequency band. Quantitatively, as shown in Table 1, the proposed matrix demonstrates a lower average insertion loss and reduced phase error across the operational band, while achieving a more compact footprint than representative matrices from recent literature. These performance improvements underscore the **wideband performance and integration advantages** of the proposed structure for millimeter-wave applications. In summary, a low gain loss (within 3 dB) with a low phase error of 1.8° at port 2 was obtained. A comparison with other similar-frequency designs is presented in Table 1. Therefore, the proposed beamforming network met the design specifications well and had a better performance than state-of-the-art devices with low loss and low profile and phase error at 26 GHz.

**Table 1.** Comparison of this work with other related research work.

Ref	Technology	Freq.	Configuration	Losses	Phase error	Size (mm ^2^) (L × W)	Bandwidth	Gain
^22^ /2021	Waveguide	28 GHz	4 × 4 BM Standard	3.4 dB	4°	≈10.71 mm	-	~15.2 dB
^23^ /2022	Microstrip	28-32 GHz	6 × 6 BM Standard	8.5 dB	10°	≈10.00 mm	4 GHz	~10–14 dB
^21^ /2023	SIW	26 GHz	4 × 4 BM Standard	5.3 dB	6.7°	≈11.54 mm	>1 GHz	>10 dB
^24^ /2024	SIW	28 GHz	4 × 4 BM Standard	6 dB	5°	≈10.71 mm	-	
^25^ /2024	SIW	26 GHz	4 × 4 BM Standard	4.8 dB	7°	≈11.54 mm	~5 GHz	
This work/2025	SIW	26 GHz	4 × 4 BM couplers	1.4 dB	1.8°	≈11.54 mm	5 GHz	9 dB

Beyond its electrical performance, the proposed SIW Butler Matrix is particularly well suited for **monolithic integration and system-level co-design**, which are essential requirements for compact millimeter-wave front-end architectures in 5G and beyond. The planar nature of SIW technology enables seamless integration with other passive and active components on a single substrate, including power dividers, filters, and transitions, while preserving low-loss wave-guiding behavior.

A key advantage of the proposed structure is its **direct compatibility with planar antenna arrays**, such as SIW-fed slot arrays and substrate-integrated patch arrays. Since both the Butler Matrix and the antenna elements can be realized using the same fabrication process and substrate stack-up, the proposed design facilitates compact beamforming modules without the need for complex interconnects or vertical transitions. This compatibility significantly reduces assembly complexity, parasitic effects, and overall system footprint, which are critical constraints at millimeter-wave frequencies.

Furthermore, SIW technology offers promising compatibility with **photonic and electro-optic circuits**, particularly in emerging hybrid microwave–photonic front-end systems. The well-defined electromagnetic confinement and planar interfaces of SIW structures simplify the integration of optoelectronic components, such as modulators and photodetectors, enabling efficient RF-to-photonic signal routing. This makes SIW-based beamforming networks attractive candidates for future high-frequency systems that combine electronic beamforming with photonic signal distribution to achieve wide bandwidth, low latency, and high integration density.

Consequently, the proposed SIW Butler Matrix is not only a wideband and compact beamforming solution at the circuit level, but also a scalable building block for **integrated, multi-functional front-end modules** targeting advanced 5G and next-generation wireless systems.

Although this work demonstrates a 4×4 Butler Matrix as a proof of concept, the proposed SIW architecture is inherently **scalable to higher-order matrices**, such as 8×8 configurations. In principle, the extension follows the same hierarchical network structure used in conventional Butler matrices, where additional SIW hybrid couplers are cascaded to generate the required phase progression and output ports.

No fundamental limitations prevent scaling the proposed crossover-free and phase-shifter-free topology to larger matrices. However, practical considerations arise as the matrix order increases. These include increased layout complexity, longer signal paths leading to higher cumulative insertion loss, and tighter control of phase balance due to phase accumulation across multiple cascaded couplers. At millimeter-wave frequencies, fabrication tolerances and via-placement accuracy may also impose constraints on achievable phase uniformity in large-scale implementations.

Despite these practical challenges, SIW technology remains well suited for higher-order Butler matrices due to its low-loss waveguiding characteristics and planar layout flexibility. With careful layout optimization, symmetry preservation, and loss balancing, larger matrices such as 8×8 can be realized while maintaining acceptable wideband performance. Therefore, the proposed architecture provides a scalable foundation for higher-order beamforming networks, particularly in applications where compactness and integration are prioritized.

## Conclusion

A 4 × 4 Butler beamforming matrix with a low-loss wideband at 26 GHz for 5G mobile proposed to produce one type of 4 × 4 application is shown to produce a distinct sequential phase difference available in the output ports. The fabricated 4 × 4 Butler matrix was designed with four 3-dB couplers to achieve substantial size reduction. There is a good agreement between the measurement and simulation results to verify the final performance of the design. A high performance 1.4 dB low loss magnitude error, 1.8° phase error and a 2.8 GHz 1 dB bandwidth are achieved. The suggested 4 × 4 antenna Butler matrix has a novel method for BFN designs for 26 GHz 5G applications along with the presented properties.

## Data Availability

Zenodo. Experimental data for “Butler Matrix Based on Substrate Integrated Waveguide Without Crossover and Phase Shifter for Millimeter Waves Applications”. DOI: https://doi.org/10.5281/zenodo.17076167. ^34^ This project contains the following underlying data:
• Experimental Data (Nawras, Coupler and BM).xlsx Data are available under the terms of the Creative Commons Attribution 4.0 International license (CC-BY 4.0).

## References

[1] Ojaroudiparchin N Shen M Zhang S : A Switchable 3-D-Coverage-Phased Array Antenna Package for 5G Mobile Terminals. *IEEE Antennas Wirel. Propag. Lett.* 2016;15(c):1747–1750. 10.1109/LAWP.2016.2532607

[2] Moon SM Yun S Yom IB : Phased Array Shaped-Beam Satellite Antenna with Boosted-Beam Control. *IEEE Trans. Antennas Propag.* 2019;67(12):7633–7636. 10.1109/TAP.2019.2930129

[3] Vallappil AK Rahim MKA Khawaja BA : Butler Matrix Based Beamforming Net-works For Phased Array Antenna Systems: A Comprehensive Review and Future Directions For 5G Applications. *IEEE Access.* 2020;9:3970–3987. 10.1109/ACCESS.2020.3047696

[4] Dyab WM Sakr AA Wu K : Dually-Polarized Butler Matrix for Base Stations With Polarization Diversity. *IEEE Trans. Microw. Theory Tech.* 2018;66(12):5543–5553. 10.1109/TMTT.2018.2880786

[5] Tsokos C : Analysis of a Multibeam Optical Beamforming Network Based on Blass Matrix Architecture. *J. Lightwave Technol.* 2018;36(16):3354–3372. 10.1109/JLT.2018.2841861

[6] Fonseca NJG : Printed S-band 4 × 4 nolen matrix for multiple beam antenna applications. *IEEE Trans. Antennas Propag.* 2009;57(6):1673–1678. 10.1109/TAP.2009.2019919

[7] Ren B Arigong M Zhou JD : A Novel Design of 4 × 4 Butler Matrix With Relatively Flexible Phase Differences. *IEEE Antennas Wirel. Propag. Lett.* 2016;15:1277–1280. 10.1109/LAWP.2015.2504719

[8] Wang Y Ma K Jian Z : A low-loss butler matrix using patch element and honeycomb concept on SISL platform. *IEEE Trans. Microw. Theory Tech.* 2018;66(8):3622–3631. 10.1109/TMTT.2018.2845868

[9] Rezvani M Nikmehr S Ghanbari L : A novel Butler matrix feeding system for BST multi-band antennas. *IEICE Electron. Express.* 2013;10(2):1–6. 10.1587/elex.10.20120606

[10] Ibrahim SZ Bialkowski ME : Wideband butler matrix in microstrip-slot technology. *APMC 2009 - Asia Pacific Microw. Conf. 2009.* 2009; pp.2104–2107.

[11] Karimbu Vallappil A Rahim MKA Khawaja BA : Compact Metamaterial Based 4 × 4 Butler Matrix with Improved Bandwidth for 5G Applications. *IEEE Access.* 2020;8:13573–13583. 10.1109/ACCESS.2020.2966125

[12] Hsieh CH Lin YT Jhan HC : Application of a two-dimensional butler matrix antenna array for tile-based beamforming. *IEICE Electron. Express.* 2019;16(11):1–6. 10.1587/elex.16.20190272

[13] Idris M : A Microstrip based RF Filter for Biosensor Applications.Ph.D. dissertation, Newcastle University, Newcastle upon Tyne, U.K.2021.

[14] Nahar T Rawat S : Efficiency enhancement techniques of microwave and millimeter‐wave antennas for 5G communication: A survey. *Trans. Emerg. Telecommun. Technol.* May 2022;33(9).

[15] Biurrun-Quel C Teniente J del-Río C : Reduced Loss and Prevention of Substrate Modes with a Novel Coplanar Waveguide Based on Gap Waveguide Technology. *Sensors.* Mar 2023;23(6). 10.3390/s23062909 PMC10052472 36991620

[16] Daneshmand M : Multi-Port RF MEMS Switches and Switch Matrices.Ph.D. dissertation, Dept. Elect. Comput. Eng., University of Waterloo, Waterloo, ON, Canada.2006.

[17] Li H : Microwave photonic dispersion immune self-interference cancellation scheme for a radio over fiber based in-band full duplex communication link. *J. Opt. Commun. Netw.* 2023;15(9):579–587. 10.1364/JOCN.488165

[18] Bozzi M Georgiadis A Wu K : Review of substrate-integrated waveguide circuits and antennas. *IET Microw. Antennas Propag.* Jun 2011;5(8):909–920. 10.1049/iet-map.2010.0463

[19] Tajik Shafiei Alavijeh A Fakharzadeh M : Asymmetrical 4×4 butler matrix and its application for single layer 8×8 butler matrix. *IEEE Trans. Antennas Propag.* 2019;67(8):5372–5379. 10.1109/TAP.2019.2916695

[20] Shao Q Chen FC Chu QX : Novel Filtering 180° Hybrid Coupler and Its Application to 2 × 4 Filtering Butler Matrix. *IEEE Trans. Microw. Theory Tech.* 2018;66(7):3288–3296. 10.1109/TMTT.2018.2829894

[21] Cao Y Chin KS Che W : A Compact 38 GHz Multibeam Antenna Array with Multifolded Butler Matrix for 5G Applications. *IEEE Antennas Wirel. Propag. Lett.* 2017;16(c):2996–2999. 10.1109/LAWP.2017.2757045

[22] Lian JW Ban YL Xiao C : Compact Substrate-Integrated 4 × 8 Butler Matrix with Sidelobe Suppression for Millimeter-Wave Multibeam Application. *IEEE Antennas Wirel. Propag. Lett.* 2018;17(5):928–932. 10.1109/LAWP.2018.2825367

[23] Rahimi R Shahbazpanahi S : A Two-Way Network Beamforming Approach Based on Total Power Minimization with Symmetric Relay Beamforming Matrices. *IEEE Access.* 2017;5:12458–12474. 10.1109/ACCESS.2017.2710908

[24] Djerafi T Fonseca NJG Wu K : Architecture and implementation of planar 4 ×4 Ku-Band nolen matrix using SIW technology. *Proc. 2008 Asia Pacific Microw. Conf. APMC 2008.* 2008; pp.4–7.

[25] Fonseca NJG Ferrando N : Nolen matrix with tapered amplitude law for linear arrays with reduced side lobe level. *Proceedings of the Fourth European Conference on Antennas and Propagation.* 2010; pp.1–5.

[26] Sivasundarapandian Suriyakala CD : A new multi-band multiple beam forming nolen matrix antenna feeding network for cognitive radio. *Int. J. Eng. Technol.* 2014;6(5):2061–2069.

[27] Hussein YM Rahim MKA Murad NA : Low Loss Wideband 4×4 Butler Matrix Networks Based on Substrate Integrated Waveguide for 5G Applications. *IEEE Access.* 2024;12:7896–7910.

[28] Almeshehe MW : Low loss waveguide-based Butler matrix with iris coupling control method for millimeterwave applications. *Waves Random Complex Media.* Feb. 2021;33:372–392. 10.1080/17455030.2021.1880032

[29] Pezhman MM Heidari A-A Ghafoorzadeh-Yazdi A : A novel single layer SIW 6 × 6 beamforming network for 5G applications. *AEU-Int. J. Electron. C.* Oct. 2022;155:154380. 10.1016/j.aeue.2022.154380

[30] Deng J-Y Zhang Y Lin W : Compact Multibeam Antenna Array Facilitated by Miniaturized Slow Wave Substrate Integrated Waveguide Butler Matrix. *IEEE Trans. Antennas Propag.* Jan. 2024;72:9561–9564. 10.1109/TAP.2024.3463203

[31] Idury SK Delmonte N Silvestri L : Design and realization of a compact substrate inte-grated coaxial line butler matrix for beamforming applications. *Int. J. Microw. Wirel. Technol.* Jan. 2024;16(5):812–818. 10.1017/S1759078723001502

[32] Stabile R Albores-Mejia A Rohit A : Integrated optical switch matrices for packet data networks. *Microsyst. Nanoeng.* 2016;2(1):1–10. 10.1017/S1759078723001502 PMC6444727 31057809

[33] Lansdowne C Hwu SU Fogleman D : Spacecraft wireless system performance degradation due to impedance mismatch in cables and connectors. *IEEE Topical Workshop on Internet of Space (TWIOS).* 2019.

[34] Liew CP : Butler Matrix Based on Substrate Integrated Waveguide Without Crossover and Phase Shifter for Millimeter Waves Applications. *Zenodo.* Sept. 2025;8. 10.5281/zenodo.17076167 PMC13264693 42299363

