# Supplementary material for: Butler Matrix Based on Substrate Integrated Waveguide Without Crossover and Phase Shifter for Millimeter Waves Applications
Source: F1000Res. 2026 Apr 24;14:969. Originally published 2025 Sep 23. [Version 2] doi: 10.12688/f1000research.168318.2 (PMC13264693; doi:10.12688/f1000research.168318.2)
Supplement: Peer review history

# Reviewer report: Reviewer response for version 2

**DOI:** 10.5256/f1000research.196449.r486521

**Published:** 2026-06-13

**Authors:** Anitha V R

**Affiliations:** 1 BMS Institute of Technology & Management, Bangalore, Karnataka, India

The manuscript addresses an important topic in millimeter-wave beamforming networks and proposes a simplified SIW-based Butler Matrix architecture. The work demonstrates practical implementation and experimental validation, which enhances its engineering relevance. The measured results show good agreement with simulations and indicate low insertion loss, low phase error, and acceptable beam steering performance.

 Major Comments

- Although the overall design procedure is presented, several critical dimensions and optimization steps are insufficiently described.
- A complete design flowchart and parameter optimization strategy should be included to improve reproducibility.
- The derivation of the coupler phase characteristics and bandwidth enhancement mechanism should be explained in greater detail.
- The manuscript does not provide complete fabrication files, detailed geometrical dimensions, or measured datasets.
- Table 1 provides comparison data, but additional metrics such as efficiency, beam scanning accuracy, and fabrication complexity could be included.
- Several grammatical issues, typographical inconsistencies, and sentence construction problems remain throughout the manuscript.

Minor Comments

1. Clearly distinguish between simulated and measured values throughout the Results section.
2. Improve figure readability by increasing font sizes and axis labels.
3. Define all abbreviations at first occurrence.
4. Ensure consistency in units and notation (GHz, dB, degrees).
5. Provide uncertainty estimates or measurement tolerances for fabricated prototypes.

Specific Recommendation

 The manuscript presents technically sound work with practical significance and experimental validation. However, improvements are required in methodological transparency, reproducibility, literature positioning, and manuscript presentation. These revisions would strengthen the scientific rigor and impact of the work.

Is the work clearly and accurately presented and does it cite the current literature?

Partly

If applicable, is the statistical analysis and its interpretation appropriate?

Not applicable

Are all the source data underlying the results available to ensure full reproducibility?

Partly

Is the study design appropriate and is the work technically sound?

Yes

Are the conclusions drawn adequately supported by the results?

Yes

Are sufficient details of methods and analysis provided to allow replication by others?

Partly

Reviewer Expertise:

Antennas, Microwaves and RF Communications

I confirm that I have read this submission and believe that I have an appropriate level of expertise to confirm that it is of an acceptable scientific standard, however I have significant reservations, as outlined above.

# Reviewer report: Reviewer response for version 2

**DOI:** 10.5256/f1000research.196449.r478745

**Published:** 2026-05-21

**Authors:** Ji-Wei Lian

**Affiliations:** 1 Nanjing University of Science and Technology, Nanjing, China

All my comments are addressed properly.  I approve of this article.

Is the work clearly and accurately presented and does it cite the current literature?

Partly

If applicable, is the statistical analysis and its interpretation appropriate?

Partly

Are all the source data underlying the results available to ensure full reproducibility?

Partly

Is the study design appropriate and is the work technically sound?

Partly

Are the conclusions drawn adequately supported by the results?

Partly

Are sufficient details of methods and analysis provided to allow replication by others?

Partly

Reviewer Expertise:

antenna

I confirm that I have read this submission and believe that I have an appropriate level of expertise to confirm that it is of an acceptable scientific standard.

# Reviewer report: Reviewer response for version 1

**DOI:** 10.5256/f1000research.185497.r442261

**Published:** 2026-01-07

**Authors:** Ji-Wei Lian

**Affiliations:** 1 Nanjing University of Science and Technology, Nanjing, China

This paper presents a 4×4 Butler matrix implemented using Substrate Integrated Waveguide (SIW) technology operating at 26 GHz, targeting millimeter-wave 5G applications. The proposed design eliminates conventional crossovers and discrete phase shifters by employing only four hybrid couplers, enhanced with interdigital structures and non-metalized vias to fine-tune performance. Both simulated and measured results are provided. There are several comments to address.

1. The revised topology of the new 4x4 Butler matrix should be provided and compared with traditional topology
2. The phase distribution of the revised topology should be thoroughly calculated and discussed to demonstrate the feasibility of this proposed Butler matrix.
3. The bandwidth is quite narrow, which is not common for SIW butler matrix. Please discuss this issue.
4. While the 4×4 case is demonstrated, it would be helpful to briefly discuss whether this architecture can be extended to larger Butler matrices (e.g., 8×8). Are there fundamental limitations in terms of layout or phase accumulation?
5. The simulated and measured results should be plotted in the same figure for comparison.
6. Fig. 11, it is not clear to see the simulated and measured results.
7. The size in table 1 should be in wavelength. The gain and bandwidth should be added.

Is the work clearly and accurately presented and does it cite the current literature?

Partly

If applicable, is the statistical analysis and its interpretation appropriate?

Partly

Are all the source data underlying the results available to ensure full reproducibility?

Partly

Is the study design appropriate and is the work technically sound?

Partly

Are the conclusions drawn adequately supported by the results?

Partly

Are sufficient details of methods and analysis provided to allow replication by others?

Partly

Reviewer Expertise:

antenna

I confirm that I have read this submission and believe that I have an appropriate level of expertise to state that I do not consider it to be of an acceptable scientific standard, for reasons outlined above.

# Response

**Published:** 2026-02-10

**Authors:** Chia Pao Liew

**Affiliations:** Energy & Chemical Engineering, Xiamen University - Malaysia, Sepang, Selangor, Malaysia

Comment 1: highlighted in the design method

 Comment 2: the following is highlighted:

 In the revised crossover-free, phase-shifter-free topology, the required phase distribution is realized through the **intrinsic phase properties of the SIW hybrid couplers** and **path-length differences** in the interconnecting SIW transmission sections.

 For an ideal 3-dB quadrature hybrid coupler:

- The two output signals have equal magnitude.
- The relative phase difference between the “through” and “coupled” ports is approximately ±90∘ \pm 90^\circ±90∘ (sign depends on port definition).

Thus, the cumulative phase at each output port can be written as a sum of:

1. hybrid-coupler phase terms, and
2. propagation phase terms along SIW paths.

* *
                                        (2)

 Where:

- *N* *_c_* = number of couplers on the signal route
- *N* *_p_* = number of SIW path segments
- *L* *_m_* = physical length of the m-th segment
- *β* *_g_* = guided propagation constant of SIW

For SIW, the guided wavenumber can be approximated by:

                                                     (3)

                                           where k₀=                    

 εᵣ is the substrate relative permittivity, and *a* *_eq_* is the equivalent SIW width determined from the via diameter and pitch. This expression allows computing the expected phase shift of any interconnect line:

*φ* *_prop_* = *β* *_g_* *L*     

 Comment 3: the following is highlighted:

 the operational bandwidth of the proposed Butler Matrix is relatively limited compared to some reported SIW-based implementations. This behaviour is primarily attributed to the **cascaded hybrid-coupler topology** employed to eliminate crossovers and discrete phase shifters. In such configurations, the overall bandwidth is governed by the **intersection of the passbands of the individual couplers**, resulting in a reduced effective bandwidth as the number of cascaded stages increases. Additionally, the bandwidth is influenced by the **phase balance requirement** intrinsic to Butler matrices. Even when acceptable impedance matching is maintained over a wider frequency range, stringent phase-error constraints impose a narrower usable bandwidth for accurate beamforming operation.

 Comment 4: the following is highlighted:

 Although this work demonstrates a 4×4 Butler Matrix as a proof of concept, the proposed SIW architecture is inherently **scalable to higher-order matrices**, such as 8×8 configurations. In principle, the extension follows the same hierarchical network structure used in conventional Butler matrices, where additional SIW hybrid couplers are cascaded to generate the required phase progression and output ports.

 No fundamental limitations prevent scaling the proposed crossover-free and phase-shifter-free topology to larger matrices. However, practical considerations arise as the matrix order increases. These include increased layout complexity, longer signal paths leading to higher cumulative insertion loss, and tighter control of phase balance due to phase accumulation across multiple cascaded couplers. At millimeter-wave frequencies, fabrication tolerances and via-placement accuracy may also impose constraints on achievable phase uniformity in large-scale implementations.

 Despite these practical challenges, SIW technology remains well suited for higher-order Butler matrices due to its low-loss waveguiding characteristics and planar layout flexibility. With careful layout optimization, symmetry preservation, and loss balancing, larger matrices such as 8×8 can be realized while maintaining acceptable broadband performance. Therefore, the proposed architecture provides a scalable foundation for higher-order beamforming networks, particularly in applications where compactness and integration are prioritized.

 Comment 5: modification was made on the figures to improve clarity with multiple lines in the Figures

 Comment 6: We have improved Fig 11.

 Comment 7: changed to wavelength

# Reviewer report: Reviewer response for version 1

**DOI:** 10.5256/f1000research.185497.r421174

**Published:** 2025-11-11

**Authors:** Giuseppe Brunetti

**Affiliations:** 1 Politecnico di Bari, Bari, Italy

The proposed Substrate Integrated Waveguide (SIW) Butler Matrix can be contextualized within the broader framework of integrated switching and beamforming networks, which are conventionally categorized into broadband and narrowband architectures. Broadband switching matrices are typically realized using distributed transmission-line elements or waveguide-based implementations, optimized to sustain phase and amplitude uniformity over wide frequency ranges.Here, my comments:

1. The manuscript lacks a clear contextualization of the proposed SIW Butler Matrix within the broader class of integrated switching matrices. A comparative discussion should be introduced early in the Introduction section, distinguishing between broadband and narrowband matrix architectures and highlighting the relevance of the proposed design in this framework.
2. The classification between broadband and narrowband switching matrices should be explicitly articulated. Broadband matrices, typically based on distributed or waveguide structures, support wide frequency operation and are suited for multi-band applications such as 5G beamforming. Conversely, narrowband architectures, often resonant or lumped-element based, provide high selectivity and low insertion loss at the expense of operational bandwidth. (see, e.g., *Butler Matrix-Based Beam Switching with Frequency-Tunable MIMO Array. In 2024 IEEE International Symposium on Antennas and Propagation and INC/USNC‐URSI Radio Science Meeting (AP-S/INC-USNC-URSI) (pp. 2475-2476). IEEE., 2024; Design of a large bandwidth 2× 2 interferometric switching cell based on a sub-wavelength grating. Journal of Optics, 23(8), 08580, 2021*).
3. The paper should include a concise review of integrated implementations of switching matrices, emphasizing microstrip, CPW, coaxial, and photonic platforms. Discussing their respective trade-offs, such as radiation losses, limited scalability, and fabrication complexity, would clarify the rationale for adopting an SIW-based solution.
4. The originality of the proposed structure should be emphasized by contrasting it with existing 4×4 Butler Matrices that rely on crossovers and phase shifters. Quantitative comparisons of insertion loss, phase error, and physical dimensions with recent literature would reinforce the claim of superior broadband performance and compactness.
5. The discussion of the SIW implementation should be expanded to underline its suitability for monolithic integration and compatibility with planar antenna arrays and photonic circuits. This aspect is particularly important for applications targeting compact, high-frequency front-end systems in 5G and beyond.

Here, my minor comments:

1. The abstract and introduction should better clarify the motivation behind eliminating crossovers and phase shifters, explaining how this contributes to both broadband performance and fabrication simplification.
2. In Table 1, the comparison with previous works could be enriched by specifying whether each listed matrix operates in the broadband or narrowband regime.
3. The terminology “wideband” and “broadband” should be used consistently throughout the text to avoid ambiguity.
4. The introduction could benefit from one or two sentences connecting SIW-based microwave matrices with analogous developments in integrated photonics, where broadband beamforming networks are being explored using similar architectural concepts.
5. Some of the numerical performance indicators (e.g., fractional bandwidth, insertion loss) should be explicitly defined or referenced to ensure clarity for readers from adjacent fields.

Is the work clearly and accurately presented and does it cite the current literature?

No

If applicable, is the statistical analysis and its interpretation appropriate?

Not applicable

Are all the source data underlying the results available to ensure full reproducibility?

No source data required

Is the study design appropriate and is the work technically sound?

Yes

Are the conclusions drawn adequately supported by the results?

No

Are sufficient details of methods and analysis provided to allow replication by others?

Yes

Reviewer Expertise:

Filter; photonics; beamforming

I confirm that I have read this submission and believe that I have an appropriate level of expertise to confirm that it is of an acceptable scientific standard, however I have significant reservations, as outlined above.

# Response

**Published:** 2026-02-10

**Authors:** Chia Pao Liew

**Affiliations:** Energy & Chemical Engineering, Xiamen University - Malaysia, Sepang, Selangor, Malaysia

Comment no. 1: the following is highlighted in the manuscript

 Beamforming networks and switching matrices are fundamental components in modern multi-antenna systems, enabling signal routing, phase control, and spatial beam steering in applications such as 5G millimeter-wave communications, radar, and satellite systems. Within this broad class, integrated switching matrices can be generally categorized into **narrowband** and **broadband** architectures, depending on their operational bandwidth, phase stability, and amplitude balance characteristics.

 Narrowband matrix architectures, including conventional waveguide and microstrip Butler matrices, are typically optimized for operation around a single center frequency. While these solutions offer compact layouts and relatively low insertion loss, their performance rapidly degrades outside the design band due to frequency-dependent phase errors and impedance mismatch. Such limitations restrict their suitability for emerging wideband and multi-carrier millimeter-wave systems.

 In contrast, broadband matrix architectures employ wideband couplers, phase shifters, or hybrid implementations to maintain stable phase progression and amplitude balance over an extended frequency range. These designs are particularly relevant for 5G and beyond systems, where wide instantaneous bandwidth, beam squint mitigation, and integration compatibility are critical design requirements. However, many reported broadband matrices rely on multilayer structures, complex transitions, or mixed technologies, which increase fabrication complexity and cost.

 In this context, Substrate Integrated Waveguide (SIW) technology provides an attractive platform for realizing broadband integrated switching matrices by combining the low-loss characteristics of conventional waveguides with the planar manufacturability of printed circuits. The proposed SIW Butler Matrix in this work is positioned within the **broadband matrix architecture class**, specifically targeting wideband operation with stable phase differences and reduced insertion loss while maintaining a fully planar and fabrication-friendly structure. By employing only SIW hybrid couplers without additional phase-shifting elements, the proposed design offers a simplified topology that directly addresses the bandwidth and integration challenges encountered in existing matrix implementations.

 Comment no. 2 - as highlighted in Comment no. 1 above

 Comment no. 3: the following is highlighted in the manuscript

 Various integrated platforms have been explored for the implementation of switching and beamforming matrices, including microstrip, coplanar waveguide (CPW), coaxial, and photonic technologies. Each platform offers distinct advantages, but also exhibits inherent limitations when applied to wideband millimeter-wave matrix architectures.

 Microstrip-based switching matrices are widely reported due to their low cost, planar form factor, and ease of integration with active components. However, at millimeter-wave frequencies, microstrip implementations suffer from increased radiation losses, surface-wave excitation, and dispersion effects, which limit achievable bandwidth and degrade phase accuracy in large matrix configurations. Coplanar waveguide (CPW) platforms alleviate some integration challenges and provide improved grounding flexibility, yet they remain susceptible to radiation leakage and conductor loss at high frequencies, particularly in dense multi-port networks.

 Coaxial and waveguide-based matrices offer superior isolation and low loss, making them attractive for high-performance systems. Nevertheless, their bulky three-dimensional structures, limited scalability, and high fabrication and assembly costs restrict their suitability for compact and highly integrated 5G and beyond applications. More recently, photonic switching matrices have been proposed to enable ultra-wide bandwidth and immunity to electromagnetic interference. Despite these advantages, photonic solutions typically require complex electro-optic conversion stages, precise alignment, and specialized fabrication processes, resulting in increased system complexity and cost.

 In comparison, Substrate Integrated Waveguide (SIW) technology combines the low-loss and high-isolation characteristics of conventional waveguides with the planar manufacturability of printed circuit technologies. This hybrid nature makes SIW particularly well suited for integrated broadband switching matrices, offering reduced radiation loss, improved phase stability, and excellent scalability while maintaining compatibility with standard PCB fabrication. Consequently, the SIW-based Butler Matrix proposed in this work provides a balanced solution that directly addresses the limitations of alternative integrated platforms.

 Comment no. 4: the following is highlighted in the manuscript

 The key contribution of this work lies in the **original structural simplicity** and performance advantages of the proposed SIW Butler Matrix compared to existing 4×4 matrix designs that rely on crossovers and discrete phase-shifter networks. Traditional implementations of 4×4 Butler Matrices in microstrip and CPW technologies often incorporate crossovers and fixed electrical-length phase shifters to achieve the required interconnections and phase progression. While functional, these elements introduce additional **insertion loss**, **phase distortion**, and **size penalties**, particularly at millimeter-wave frequencies, due to increased discontinuities, radiation effects, and dispersion.

 By contrast, the proposed SIW design employs only SIW hybrid couplers without separate crossovers or discrete phase shifters, leading to a reduction in cumulative junction loss and improved phase fidelity over a wide frequency band. Quantitatively, as shown in Table 1, the proposed matrix demonstrates a lower average insertion loss and reduced phase error across the operational band, while achieving a more compact footprint than representative matrices from recent literature. These performance improvements underscore the **broadband performance and integration advantages** of the proposed structure for millimeter-wave applications.

 Comment 5: the following is highlighted in the manuscript

 Beyond its electrical performance, the proposed SIW Butler Matrix is particularly well suited for **monolithic integration and system-level co-design**, which are essential requirements for compact millimeter-wave front-end architectures in 5G and beyond. The planar nature of SIW technology enables seamless integration with other passive and active components on a single substrate, including power dividers, filters, and transitions, while preserving low-loss wave-guiding behavior.

 A key advantage of the proposed structure is its **direct compatibility with planar antenna arrays**, such as SIW-fed slot arrays and substrate-integrated patch arrays. Since both the Butler Matrix and the antenna elements can be realized using the same fabrication process and substrate stack-up, the proposed design facilitates compact beamforming modules without the need for complex interconnects or vertical transitions. This compatibility significantly reduces assembly complexity, parasitic effects, and overall system footprint, which are critical constraints at millimeter-wave frequencies.

 Furthermore, SIW technology offers promising compatibility with **photonic and electro-optic circuits**, particularly in emerging hybrid microwave–photonic front-end systems. The well-defined electromagnetic confinement and planar interfaces of SIW structures simplify the integration of optoelectronic components, such as modulators and photodetectors, enabling efficient RF-to-photonic signal routing. This makes SIW-based beamforming networks attractive candidates for future high-frequency systems that combine electronic beamforming with photonic signal distribution to achieve wide bandwidth, low latency, and high integration density.

 Consequently, the proposed SIW Butler Matrix is not only a broadband and compact beamforming solution at the circuit level, but also a scalable building block for **integrated, multi-functional front-end modules** targeting advanced 5G and next-generation wireless systems.

 Minor comment no.1: the following is highlighted in the manuscript

 This paper presents a compact and broadband 4×4 Butler Matrix implemented using Substrate Integrated Waveguide (SIW) technology. In contrast to conventional Butler Matrices that rely on crossovers and discrete phase shifters, the proposed design employs only SIW hybrid couplers to realize the required phase progression and signal distribution. Eliminating crossovers and phase shifters significantly reduces structural discontinuities, junction losses, and frequency-dependent phase errors, thereby enhancing broadband performance. Moreover, the simplified topology minimizes fabrication complexity and improves suitability for planar and monolithic integration, making the proposed matrix well suited for compact millimeter-wave beamforming front ends in 5G and beyond systems.

 Minor comment no. 2: Considered

 Minor comment no. 3: corrected to just wideband

 Minor comment no. 4: Added as above

 Minor comment no. 5: Defined fractional bandwidth
